# Detection and transport of environmental DNA from two federally endangered mussels

**DOI:** 10.1371/journal.pone.0304323

**Published:** 2024-10-17

**Authors:** Brandon J. Sansom, Dannise V. Ruiz-Ramos, Nathan L. Thompson, Maura O. Roberts, Zachary A. Taylor, Katie Ortiz, Jess W. Jones, Catherine A. Richter, Katy E. Klymus

**Affiliations:** 1 U.S. Geological Survey–Columbia Environmental Research Center, Columbia, MO, United States of America; 2 Department of Natural Sciences, University of Maryland Eastern Shore, Princess Anne, MD, United States of America; 3 Maryland Department of Natural Resources, Annapolis, MD, United States of America; 4 Department of Fish and Wildlife Conservation, Virginia Polytechnic Institute and State University, Blacksburg, VA, United States of America; 5 Department of Fish and Wildlife Conservation, U.S. Fish and Wildlife Service, Blacksburg, VA, United States of America; University of Hyogo, JAPAN

## Abstract

Environmental DNA (eDNA) offers a novel approach to supplement traditional surveys and provide increased spatial and temporal information on species detection, and it can be especially beneficial for detecting at risk or threatened species with minimal impact on the target species. The transport of eDNA in lotic environments is an important component in providing more informed descriptions of where and when a species is present, but eDNA transport phenomena are not well understood. In this study, we used species-specific assays to detect eDNA from two federally endangered mussels in two geographically distinct rivers. Using the eDNA concentrations measured from field samples, we developed a one-dimensional (1D) hydrodynamic transport model to predict the downstream fate and transport of eDNA. We detected eDNA from both federally endangered mussels across several seasons and flow rates and up to 3.5 km downstream from the source populations, but the detection rates and eDNA concentrations were highly variable across and within rivers and study reaches. Our 1D transport models successfully integrated the variability of the eDNA field samples into the model predictions and overall model results were generally within ±1 standard error of the eDNA field concentration values. Overall, the results of this study demonstrate the importance of optimizing the spatial locations from where eDNA is collected downstream from a source population, and it highlights the need to improve understanding on the shedding mechanisms and magnitude of eDNA from source populations and biogeomorphic processes that influence eDNA transport.

## 1. Introduction

Environmental DNA analysis is a novel tool for detecting species and is now being implemented or considered for implementation in species monitoring and bio-surveillance alongside traditional survey methods in aquatic systems [1,2]. For instance, the U.S. Fish and Wildlife Service (USFWS) uses eDNA methods to monitor invasive carp in the upper Mississippi River of the United States [3] and Natural England approved the use of eDNA surveys for detecting the threatened Great Crested Newt in the United Kingdom [4]. Beyond inferring species presence based on eDNA detections, data from eDNA surveys also have the potential to infer relative abundance and spatial distribution of a population [5], which is valuable to wildlife management of aquatic threatened and endangered species.

Knowing where and when to sample for eDNA is critical for a robust sampling design, especially for studies working with threatened and endangered species. In lotic environments, eDNA is likely to be sampled and collected some distance downstream from the DNA source. Understanding the spatial and temporal distribution resulting from eDNA transport, along with relevant biological properties such as decay and sorption, is necessary to provide the most accurate information about the source of DNA. However, eDNA transport is a relatively new research area that is complicated by numerous environmental factors, and many uncertainties exist associated with the downstream transport of eDNA in lotic environments [6,7]. Several approaches have been used to predict eDNA transport. Models developed for fine particulate organic matter transport have most commonly been adopted by ascribing a first-order decay constant to eDNA, usually determined by the longitudinal loss rate measured from eDNA field samples, in order to predict the downstream movement [8–10]. A similar approach has also been applied using laboratory-derived degradation estimates in numerical advection-dispersion models to predict the concentration of eDNA at known distances downstream from the source [11–13]. These approaches however, often over-generalize important hydraulic factors, such as flow velocity or depth, into an averaged value and may be improved by including geomorphic features such as slope or roughness, or more detailed hydraulic processes [7,14]. More recently, some studies have integrated quantitative analyses with high spatial-temporal resolution of hydrodynamic models to estimate transport or species biomass and abundance [15–17].

Furthermore, there is a need to incorporate eDNA ecology studies that investigate the production, degradation, and interactions of eDNA with the environment into transport models [14,18]. Interpretation of eDNA detections relative to a population’s location or size is complex, particularly in lotic systems because water flow and hydraulics greatly influence the movement and dilution of eDNA and its subsequent detection [14]. As a result, it has been noted that eDNA is highly variable in time and space [19,20], varying with stream morphology and downstream displacement as advective forces result in a continuous dilution of an eDNA signal [21,22]. Studies have also found that processes involved in the sorption and retention of DNA in sediments and biofilms may play a major role in removal of DNA from the system [14,23]. Moreover, multiple sources of DNA distributed throughout a catchment, or target species that are highly mobile such as many riverine fish species, may further complicate the interpretation of eDNA within and along a river gradient [16,24].

Freshwater mussels make a good study organism for the use of eDNA because they are often found in dense aggregations known as mussel beds (hereafter, beds) and are relatively sedentary as adult organisms compared to other aquatic taxa such as fish. Mussels also have a unique life history where distinct reproductive events may easily be detected in eDNA samples. Male mussels release long-lived aggregates of sperm that drift downstream and female mussels filter these aggregates to fertilize their eggs [25]. Following fertilization, females display or release larvae in ways to elicit an encounter with a host fish [26], a process that also releases a large amount of genetic material into the environment. Thus, inference of field mussel eDNA detections should not be hindered by the movement of the target species, and potential spawning events may be readily captured using eDNA methods. A further noteworthy aspect of freshwater mussels is that their mitochondrial genome is inherited in from both parents, with the maternal mitochondrial genome found in somatic cells and the female germ line, and a separate paternal mitochondrial genome found in the male germ line [27]. Coupled with these distinctive aspects of mussel biology, freshwater mussels are of high conservation concern globally. Within North America roughly one-third of the nearly 300 species are federally listed in the United States and two-thirds are considered imperiled [28]. Apart from being potentially disruptive to the sampled organisms and their habitat, traditional survey techniques for freshwater mussels can involve logistically difficult and time-consuming work, including SCUBA or snorkeling. In contrast, sampling for eDNA requires less time per site and less taxonomic expertise than traditional sampling, allowing efficient prioritization of sites and expansion of scope than traditional survey efforts. Environmental DNA sampling may therefore reduce stress to threatened populations, minimize sampling time, and reduce the requirement for highly trained field personnel [29].

Here, we examined the spatial and temporal trends of eDNA from two federally endangered mussels (*Cumberlandia monodonta* and *Epioblasma capsaeformis*) in two separate rivers (Big Piney, MO and Clinch River, TN). Our goals were to a) develop a field sampling protocol to detect eDNA from two federally endangered species at fixed locations downstream from the source population, and b) use the information from field samples to develop a modeling framework to improve understanding of eDNA downstream transport. We sampled water downstream from known populations of each mussel species and quantified the amount of eDNA present in each field sample. Using the field sampled eDNA concentrations and laboratory derived eDNA decay rates, we developed a one-dimensional (1D) hydrodynamic transport model that incorporated hydraulic and biological characteristics to simulate the downstream fate and transport of eDNA.

## 2. Materials and methods

Environmental DNA field samples were collected and amplified to test for the target species in each river at known locations downstream from the source populations over two years. The field measured eDNA concentrations and physical data used to generate the hydraulic and transport models are available through several USGS ScienceBase data releases [30–33].

### 2.1. Study areas and organisms

This study was conducted in two geographically distinct rivers, the Big Piney River in Missouri, and the Clinch River in Tennessee. These rivers were selected on the basis of persistent and healthy populations of two federally endangered mussels, *Cumberlandia monodonta* in the Big Piney River and *Epioblasma capsaeformis* in the Clinch River.

#### 2.1.1. Lazy Day reach in the Big Piney River: *Cumberlandia monodonta*

**Fig 1:**
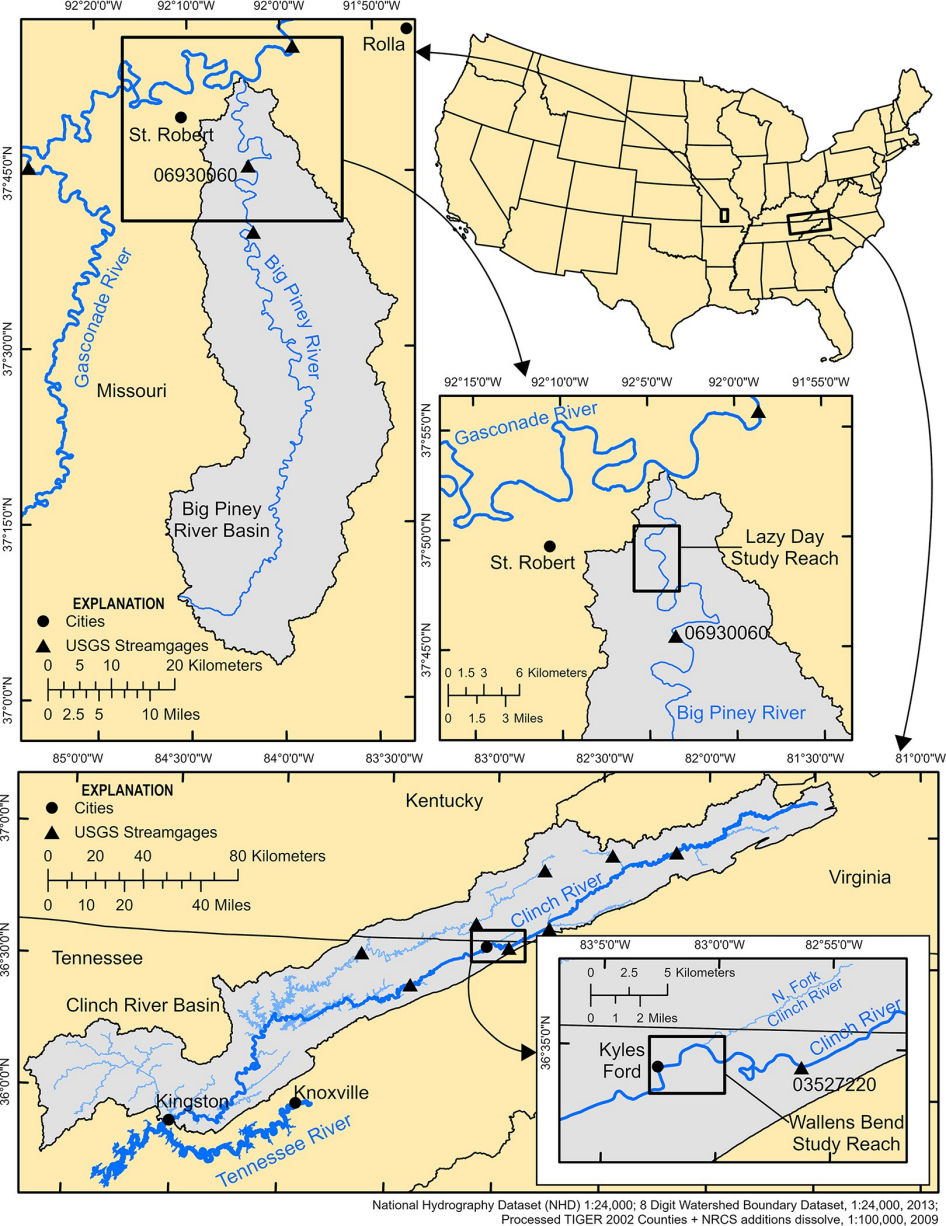
Study reach maps. Sites maps of the Lazy Day study reach located in the Big Piney River near St. Robert, MO and the Wallens Bend study reach located in the Clinch River near Kyles Ford, TN.

The Big Piney River is part of the Missouri River watershed and is located in the Ozark Highlands region. It is largely spring fed and originates near Cabool, MO, flows 177 km northeast and drains into the Gasconade River near St. Robert, MO. The study reach in the Big Piney River was located at the Lazy Day reach (hereafter Lazy Day; Fig 1) and focused on a mussel bed primarily composed of a single species, *C*. *monodonta*. *Cumberlandia monodonta* is a federally endangered mussel that occurs in the Mississippi River Basin and inhabits medium to large rivers. The study reach at Lazy Day began 0.1 km upstream from the mussel bed and extended 2.2 km downstream from the mussel bed. Private landowner access was granted to access sample sites throughout the Lazy Day reach.

#### 2.1.2. Wallens Bend reach in the Clinch River: *Epioblasma capsaeformis*

The Clinch River is part of the Ohio River watershed and is located in the Appalachian Mountains. It originates near Tazewell, Virginia, flows nearly 483 km southwest and drains into the Tennessee River near Kingston, TN (Fig 1). The study reach in the Clinch River was located at the Wallens Bend reach (hereafter Wallens Bend) near Kyles Ford, TN, where a large, multispecies, and dense mussel bed is present, which includes a large population of the federally endangered *E*. *capsaeformis*. *Epioblasma capsaeformis* is endemic to the Tennessee and Cumberland River drainages of the Mississippi River Basin and is generally found in riffle habitat in small to large rivers. The study reach at Wallens Bend began 0.1 km upstream from the mussel bed and extended 4.4 km downstream from the mussel bed. The North Fork of the Clinch River (hereafter North Fork) drains into the Clinch River 0.6 km downstream from the mussel bed and contributes around 5 percent of the Clinch River’s mean annual discharge. To account for the water flow and eDNA contributed by the North Fork, we also included a small portion (0.1 km) of the North Fork upstream from the confluence with the Clinch River in the study reach. Site access at Wallens Bend was granted by Tennessee Wildlife Resources and The Nature Conservancy.

### 2.2. eDNA samples

#### 2.2.1. eDNA field collection

Environmental DNA samples were collected at Lazy Day in the Big Piney River, MO, and Wallens Bend in the Clinch River, TN. At each study reach, we placed sample stations throughout the reach to capture spatial variability of eDNA transport. These sampling stations were located at 100 m upstream from the mussel bed, at the bottom of the bed, 100 m downstream from the bottom of the bed, 500 m downstream from the bed, 1000 m downstream from the bed, and either 2000 m downstream from the bed for the Lazy Day reach or 3500 m downstream from the bed for Wallens Bend. An additional transect at 800 m downstream was also included for the Lazy Day reach. At Lazy Day, field sampling began at the 500 m downstream station and proceeded upstream to the 100 m upstream station, followed by the 1000 m and the 2000 m downstream stations. All samples were collected from the left descending bank. On three separate sampling events, mid-channel samples were also collected at each sampling station at Lazy Day. The mid-channel sample location was accessed either by wading or from a boat. The mid-channel samples were in addition to the samples collected from the left descending bank and used to evaluate the spatial heterogeneity of eDNA at a sampling location. At Wallens Bend, field sampling began at the 3500 m station and moved upstream to the station 1000 m downstream from the bed. For both of these stations, samples were taken from the right descending bank. The field crew then proceeded to the station 100 m upstream from the bed and continued sampling downstream ending at the station 500 m downstream from the bed. These samples were collected from the left descending bank. This sampling design for both rivers was designed around access constraints along each river reach and to minimize potential contamination traveling between sampling stations.

At each sampling station, 1 L of DI water field blank was filtered using a Smith-Root eDNA sampler (Smith-Root, Vancouver, WA), and four eDNA samples of 1 L each were filtered using a Smith-Root eDNA sampler fitted with a 4-filter trident pole sampler. Filter packs were loaded with a PES filter membrane with a pore size of 1.2 μm. Depending on site conditions, filtering took 30 to 40 minutes per station, for a total of 6 to 7 hours per study reach. After filtration, filter membranes were stored in 100% ethanol before moving to the next station. Self-preserving filter packs were used for the samples from Wallens Bend. All personnel handling eDNA samples wore sterile nitrile gloves, which were changed between each sample.

#### 2.2.2. eDNA sample processing

2.2.2.1. *eDNA field samples*. In the laboratory, filters were taken out of the ethanol and placed in new 1.5 mL tubes to dry overnight in a biological safety cabinet. The filters were transferred to 5 ml PowerWater DNA Bead Tubes (Qiagen, Venlo, Netherlands) with 1 mL of MDT buffer and vortexed horizontally at maximum speed for 5 minutes. After vortexing, the buffer was left to settle for 5–10 minutes, then transferred to a new 1.5 mL tube and centrifuged at 13,000 g for one minute. 180 μL of the MDT buffer was transferred to a new 1.5 mL tube, and 20 μL of proteinase K was added. The samples were then extracted on a QuickGene-Auto12S extraction robot (ADS Biotec, Ohaha, NE) following the QuickGene AutoS Tissue DNA extraction kit (ADS Biotec, Ohaha, NE) protocol and eluted in 50 μL CDT elution buffer.

2.2.2.2. *Assay design and testing*. Samples from Lazy Day were amplified with qPCR assays for *C*. *monodonta*. For *C*. *monodonta*, we used a published COI qPCR assay [34] as an alternative binding site in the Cmon-gBlock2 (Integrated DNA Technologies (ITD), Inc., Coralville, IA). Samples for Wallens Bend were amplified with qPCR assays for *E*. *capsaeformis*. For the *E*. *capsaeformis* assay design, published CO1 sequences of female *E*. *capsaeformis* (AY094372.1- AY094374.1, AY654996.1) were used to design qPCR assays with IDT’s PrimerQuest tool. Further details for each assay can be found in Table A in S1 Text. The specificity and sensitivity of the assays were tested against DNA extractions from other sympatric freshwater mussels’ species [Tables A and B in S1 Text; 35]

For sensitivity testing of the assays, a gBlock including the target sequence was used as a standard. The limits of detection (LOD) and quantification (LOQ) were measured for the *C*. *monodonta* and *E*. *capsaeformis* assays by making six 4-fold serial dilutions of the synthetic DNA standard from approximately 0.25 copies/reaction to 1024 copies/reaction. Twenty-four replicates per standard dilution were amplified with the standard conditions, and the LOD and LOQ were calculated with a LOD/LOQ calculator R script [36]. LOD was defined as the lowest initial concentration with 95% positive replicates, LOQ as the lowest standard concentration quantified with a coefficient of variation (CV) less than 35% [35]. Environmental DNA samples were blocked across sampling events and analyzed in triplicate on a 96-well PCR plate, with standards and three negative controls on each plate. For each assay, we used the following conditions: 2 or 3 μL samples in 20 μL total reaction volume with 500 nM forward primer, 500 nM reverse primer, 125 nM probe, 125 nM HemT forward and reverse primers, 94 nM HemT probe, 5400 copies internal positive control (IPC), and 1X Environmental Master Mix (Applied Biosystems, Waltham, MA) with PCR conditions of 95°C for 10 minutes start, and 40 cycles of 95°C for 15 seconds, 60°C for 1 minute. A plate read occurred at the end of each cycle to measure the reporter probe fluorescence for DNA quantification.

2.2.2.3. *eDNA qPCR analyses*. Quantitative PCR data were exported from the CFX96 Touch Real-Time PCR Detection System (Bio-Rad Laboratories, Inc., Hercules, CA) machine and checked for errors. Quality control of qPCR was performed by checking that the assay’s standard performance fell within acceptable ranges (efficiency of 90% to 110%, R2 > 0.98, and slope -3.2 to -3.5), as well as amplification of the gDNA controls and IPC controls. Averages of copies/reaction for the technical replicates from each water sample were converted to copies/L. A sample was inhibited if the Cq of the IPC in the sample delayed by more than 3 cycles relative to the mean Cq of the IPCs in the NTCs in that qPCR plate. Inhibited samples were re-run with a 1:10 DNA to water dilution. If the mean Cq value of the IPC from the diluted sample was less than the IPC mean Cq value from the undiluted sample, the copy number value from the diluted sample was used in subsequent analysis. Amplification was defined as any exponential amplification before 40 cycles, in at least one out of three PCR technical replicates. Percent amplification was defined as number of positive amplifications out of the total PCR technical replicates for that water sample. The percentage detection was calculated as number of samples at a station that amplified divided by the total number of samples taken at that station during the sampling event.

### 2.3. Physical habitat data

At each study reach, we collected physical habitat and environmental data to better understand the environmental and abiotic drivers to eDNA concentration and transport. Environmental variables were sampled in succession with field eDNA samples. Detailed topography, bathymetry, and hydraulic surveys were conducted to further characterize and quantify habitat in each study reach. A brief overview to the data collection and processing methods are provided below, while a comprehensive description on data collection and processing are provided in Supporting Information 1C: One-dimensional hydraulic model development.

#### 2.3.1. Physical habitat data collection

Water temperature, specific conductivity, pH, and dissolved oxygen were measured at each sampling station at the time eDNA samples were taken. Bathymetry, topography, and hydraulic data were collected to encompass the entire study reach in both rivers. These survey methods used real-time kinematic (RTK) global navigation satellite system (GNSS) with a single base. At each study reach we collected stream bathymetry along planned transects using a single-beam echosounder (CEEPULSE 100 series, CEE HydroSystems, New South Wales, Australia) in areas where we were able to operate a remotely operated boat (CEE HydroSystems CEE-USV, New South Wales, Australia) and also collected additional bathymetry and topographic data in shallow or vegetated areas using a Trimble TSC3 (Trimble Inc., Westminster, CO) handheld data controller. Aerial lidar for the study areas was also obtained from state GIS repositories to provide floodplain elevation data. At the Lazy Day reach, we also supplemented bathymetry, topographic, and aerial lidar data with terrestrial lidar to capture the elevation of exposed banks and bars using a boat mounted lidar system (Velodyne lidar Puck LITE, Velodyne Lidar, San Jose, CA). Hydraulic data were collected at a range of discharges observed throughout the eDNA sampling time frame. Velocity data in each study reach were collected using a 600-kilohertz RiverRay acoustic Doppler current profiler (ADCP; Teledyne RD Instruments, Poway, CA) and followed general survey procedures to quantify mean discharge [37]. Supplemental ADCP transects were also driven at various locations through each study reach to provide velocity data for model evaluation (see below). Finally, a water-surface elevation profile was collected for every discharge measurement. Position and water-surface elevation data were measured along the length of the study reach using a Trimble R2 GNSS receiver (Trimble Inc., Westminster, CO) mounted on a boat or survey rod. Additional water-surface elevation data were provided by temporary stream gaging stations (gages) installed within the study reaches for the duration of hydraulic data collection. Water level was logged every 15 minutes using an Onset HOBO MX2001 Water Level Data Logger installed in each gage.

#### 2.3.2. Physical habitat data processing

Topography, bathymetry, and terrestrial lidar data were edited in Hypack 2018–2020 (Xylem, Middletown, CT) to remove erroneous elevation points, points if the GNSS solution was not fixed, or points that appeared to represent non-ground surfaces such as vegetation. A continuous digital elevation model (DEM) was created by merging the bathymetry, topography, and terrestrial lidar data collected at each study site. Merged datasets were converted to shapefile format and combined to create a triangulated irregular network (TIN) using Delaunay conforming triangulation. Soft breaklines were incorporated at the dataset boundaries to prevent artifacts between different datasets. TINs were manually edited based on expert opinion to remove artifacts caused by spatial gaps in data. These TINs were converted to a TIFF raster using natural neighbors interpolation, with a resolution of 2 m for both study reach DEMs.

Boat-collected water-surface elevation data were edited in Hypack 2018–2020 (Xylem, Middletown, CT) using the single-beam editor to remove erroneous and non-fixed position solution points. These edited data and the manual RTK water-surface data were converted to shapefile format and snapped to a stream centerline to determine the streamwise location of each point. Dense water-surface profiles (>1000 points) were thinned to one point per meter of streamwise distance, using the median value. Water-surface elevation profiles were supplemented with water-surface elevations extracted from the temporary gage records, using the closest timestamp for each discharge measurement.

ADCP data were processed using WinRiver II Software (Teledyne RD Instruments, Poway, CA) and used for model calibration and evaluation (see below). The computed average discharge with the lowest transect discharge errors was used as the final calibration discharge, and the computed average velocity data were used as evaluation data to evaluate the 1D model’s performance. ADCP measurements from different locations at Wallens Bend were used to compute discharge for either the North Fork or the portion of the Clinch reach upstream from the tributary, depending on where measurements were collected that day. ADCP transect data were also converted to shapefile format and snapped to a stream centerline to determine the streamwise location of each set of transect measurements.

### 2.4. Predicting eDNA downstream transport

The downstream transport of eDNA from each mussel bed was predicted using a 1D hydraulic transport model. A hydraulic model was calibrated and validated using field collected bathymetry, topography, and hydraulic data. The fate and transport of eDNA was predicted by using the calibration hydraulic solution and incorporating a laboratory derived biological decay rate constant to account for biological degradation over time. The general overview of the model development is provided below, while the specific details including the number and spacing of cross sections, reach length, specific configurations, model parameters and step-by-step procedures for each reach can be found in Supporting Information 3: One-dimensional hydraulic model development.

#### 2.4.1. One-dimensional hydraulic transport model

A 1D hydraulic model was created for each study site and was developed using the HEC-GeoRAS 10.5 extension [38] in ArcGIS Desktop 10.7.1 using a stream centerline and spaced cross sections along the entire model reach. For all model cross-sections, elevation data were extracted along the cross-section line from the study reach DEM. Hydraulic models were configured and run at steady flow using HEC-RAS 5.0.7 [39]. HEC-RAS predicts the velocity and water depth of a given cross section using the energy equation [40]:

$$Y_{2}+Z_{2}+\frac{\alpha_{2}V_{2}^{2}}{2g}=Y_{1}+Z_{1}+\frac{\alpha_{1}V_{1}^{2}}{2g}+h_{e}$$ (1)

where *Y* is the water depth, *Z* is the channel elevation, *V* is the cross-sectional average velocity, *α* is a velocity weighting coefficient, *h_e_* is the energy head loss, *g* is the gravitation acceleration, and subscripts 1 and 2 denote cross sections 1 and 2, respectively. HEC-RAS model runs were performed with a custom script in Python 3.8.12 [41], using rascontrol version 0.11 [42] to automate HEC-RAS simulations [43].

Our hydraulic modeling approach assumes that the study reaches did not experience appreciable topographic change from erosion and deposition during the period of investigation and over the range of simulated discharges. Usage of steady flow modeling indicates the assumption that discharge did not vary substantially during the eDNA sampling events.

Model calibration was performed for the range of calibration discharges using the discharge and water-surface profile data collected in the field. The purpose of calibration is to determine the appropriate model conditions for the simulation discharges, the discharges of interest that (in most cases) were not measured in the field. The calibrated model conditions were downstream slope (used for the normal depth boundary condition) and Manning’s roughness (Manning’s *n*) within the active channel. The Wallens Bend model also uses upstream water-surface elevations in the Clinch and North Fork as boundary conditions.

These calibrated values for the downstream slope and Manning’s *n*, and the measured values of upstream water-surface elevation (Wallens Bend only) were used to create a regression relationship between discharge and each parameter. These curves were generated by fitting power law or linear functions to the parameter values to find the optimal fit. These best fit regressions yield an optimized parameter value for each discharge value to be simulated.

We simulated flows that corresponded to the discharge within each study reach for unique species-discharge combinations for which sufficient field data were available for model comparison. By sufficiency we mean unique species-discharge combinations within each river where a measurable amount of eDNA was quantified at either a) the upstream extent of the study reach or at the bottom of the mussel bed to provide a boundary condition for the model and b) measurable eDNA detections were observed downstream to evaluate model results. This resulted in a total of 5 unique eDNA sampling events (2 flows at Lazy Day and 3 flows at Wallens Bend; Table 1). Discharges within the study reach were calculated using the average ratio of the ADCP-measured discharges to the discharges recorded at the closest U.S. Geological Survey streamgage (LD, USGS 06930060 Big Piney below Fort Leonard Wood, MO; WB, USGS 03527220 Clinch River near Looneys Gap, TN) at the time of ADCP discharge measurements. To obtain the estimated discharge in the study reach for each day of eDNA sampling, this average ratio was multiplied by the U.S. Geological Survey streamgage discharge at the time of each eDNA sample collection. These estimated discharges were then averaged for each eDNA sampling day to get a single discharge for that day. The estimated flow rates for Wallens Bend correspond to the discharge for the section downstream from the North Fork tributary. The tributary discharge was estimated as 5.5% of the main stem discharge downstream from the tributary junction. The main stem discharge upstream from the tributary was computed from the difference of these two flow rates.

**Table 1:** Transport model inputs and parameters. List of dates, study reach, discharge values, and the initial eDNA concentration at the upstream boundary and decay rate used in the eDNA transport model for *Cumberlandia monodonta* at the Lazy Day reach in the Big Piney River, MO and *Epioblasma capsaeformis* at the Wallens Bend reach in the Clinch River, TN.

Date	Study Reach	Discharge (m^3^/s)	Mean Initial Condition		Upper Error Estimate		Lower Error Estimate	
			Upstream Boundary Concentration (copies/L)	Decay Rate (day^-1^)	Upstream Boundary Concentration (copies/L)	Decay Rate (day^-1^)	Upstream Boundary Concentration (copies/L)	Decay Rate (day^-1^)
20200707	Lazy Day	16.26	197.2	1.6	343.5	1.3	50.9	1.9
20210723	Lazy Day	11.81	28.2	1.6	77.1	1.3	*	*
20200914	Wallens Bend	16.41	184.2	1.8	257.0	1.4	111.4	2.2
20210810	Wallens Bend	7.31	180.0	1.8	201.1	1.4	158.9	2.2
20210908	Wallens Bend	15.16	82.4	1.8	101.5	1.4	63.3	2.2

*The mean upstream boundary condition minus 1 standard error of the qPCR triplicate was negative for the upstream condition on 20210723. Therefore, the lower error estimate was assumed to be zero throughout the study reach.

Each simulation was run using the calibration curve values for the downstream slope and Manning’s *n* for each of the simulation discharges. Sensitivity analysis simulations were also performed using Manning’s *n* values at 85 and 115% of the optimum calibrated Manning’s *n*. These sensitivity analyses were run for each of the simulation discharges to determine if varying the Manning’s *n* calibration by ± 15% had an appreciable effect on the simulated water-surface profiles and velocities.

#### 2.4.2. eDNA transport modeling

For each of the 5 unique eDNA sampling events, we simulated 1D eDNA transport using the hydraulic solution from above. We designated eDNA as an arbitrary constituent using the water quality module in HEC-RAS 5.0.7 [39]. HEC-RAS uses the law of conservation of mass and simulates transport and diffusion processes as:

$$\frac{\delta C}{\delta t}+V\frac{\delta C}{\delta x}=D\frac{\delta^{2}C}{\delta x^{2}}-kC$$ (2)

where *C* is the arbitrary constituent concentration, *t* is the simulation time, *x* is the flow distance, *D* is the longitudinal dispersion coefficient, and *k* is the first-order eDNA decay rate constant (see Table 1 for *k* values used). The longitudinal dispersion coefficient was computed within the model as:

$$D=m*0.011\frac{V^{2}w^{2}}{Yu^{*}}$$ (3)

where *m* is a user assigned multiplier, *w* is the average channel width, and u^*^ is the shear velocity which is calculated as:

$$u^{*}=\sqrt{gYS}$$ (4)

where *S* is the friction slope.

The upstream boundary condition for each simulation was set to reflect the eDNA concentration of the respective field sample and was typically set as the field eDNA concentration sampled at the bottom of the mussel bed. However, a higher field eDNA concentration was observed either at the 100 m upstream or 100 m downstream sampling location for two unique sampling events, and this value was used as the upstream boundary condition (Lazy Day: *C*. *monodonta* 7/7/20–100 m downstream; Wallens Bend: *E*. *capsaeformis* 9/8/2021–100 m upstream). To account for the variation of the eDNA field samples at the upstream boundary condition, as well as the laboratory derived decay constants, we ran three model simulations for each of the 5 unique eDNA sampling events: a) the mean eDNA concentration at the upstream boundary with the mean *k* (hereafter the ‘mean initial condition’), b) the mean eDNA concentration at the upstream boundary plus 1 standard error (SE) with the mean *k* minus 1 SE (hereafter the ‘upper error estimate’), and c) the mean eDNA concentration at the upstream boundary minus 1 SE with the mean *k* plus 1 SE (hereafter the ‘lower error estimate’, Table 1). For Wallens Bend, the North Fork was set to have a zero-concentration boundary condition as no target mussels are found in the tributary. All other boundaries and water quality cells were set to have a zero-concentration initial condition. Simulations were run for a 24-hr time period to ensure the model reached a steady-state concentration throughout the entire study reach.

### 2.5. Statistical analyses

A linear regression analysis was performed between eDNA concentration and environmental variables to determine if certain environmental parameters influenced the eDNA signal. Because of the high variability of eDNA concentration within each sampling event and wanting to compare the strongest eDNA signal to each environmental variable, we only used the maximum concentration of eDNA at each sampling event, regardless of where the location of the maximum concentration occurred. Independent ordinary least squares regressions were performed between eDNA and discharge, water temperature, conductivity, and pH at each study reach.

## 3. Results

### 3.1. eDNA assay design

The *C*. *monodonta* COI2 assay was tested against 19 sympatric species (Table B in S1 Text) none of which amplified. The LOD for the assay when using 3 sample replicates was 1.4 copies/reaction, and the LOQ was 18 copies/reaction. The *E*. *capsaeformis* COI3 assay amplified the DNA extracts of 2 of the 32 sympatric species that were tested (Table B in S1 Text). Both species that amplified (*Epioblasma brevidens* [Lea, 1831] and *Epioblasma aureola* [Jones and Neves, 2010]) are closely related to *E*. *capsaeformis*. *Epioblasma aureola* is not present at Wallens Bend and the nearest population only has a few dozen individuals remaining and is over 200 km upstream. *Epioblasma brevidens* is present at Wallens bend, but not in densities as high as *E*. *capsaeformis*. One swab sample from *Ortmanniana pectorosa* (Conrad, 1834) amplified in the late cycle (Cq 39), but we could not recover a sequence from the qPCR product. The efficiency of the assay ranged between 98–110% for the specificity tests. The LOD for the *E*. *capsaeformis* assay when using 3 sample replicates was 1.4 copies/reaction, and the LOQ was 12 copies/reaction. Furthermore, the assay has successfully detected *C*. *monodonta* eDNA from laboratory experiments [44].

### 3.2. eDNA field samples

**Fig 2:**
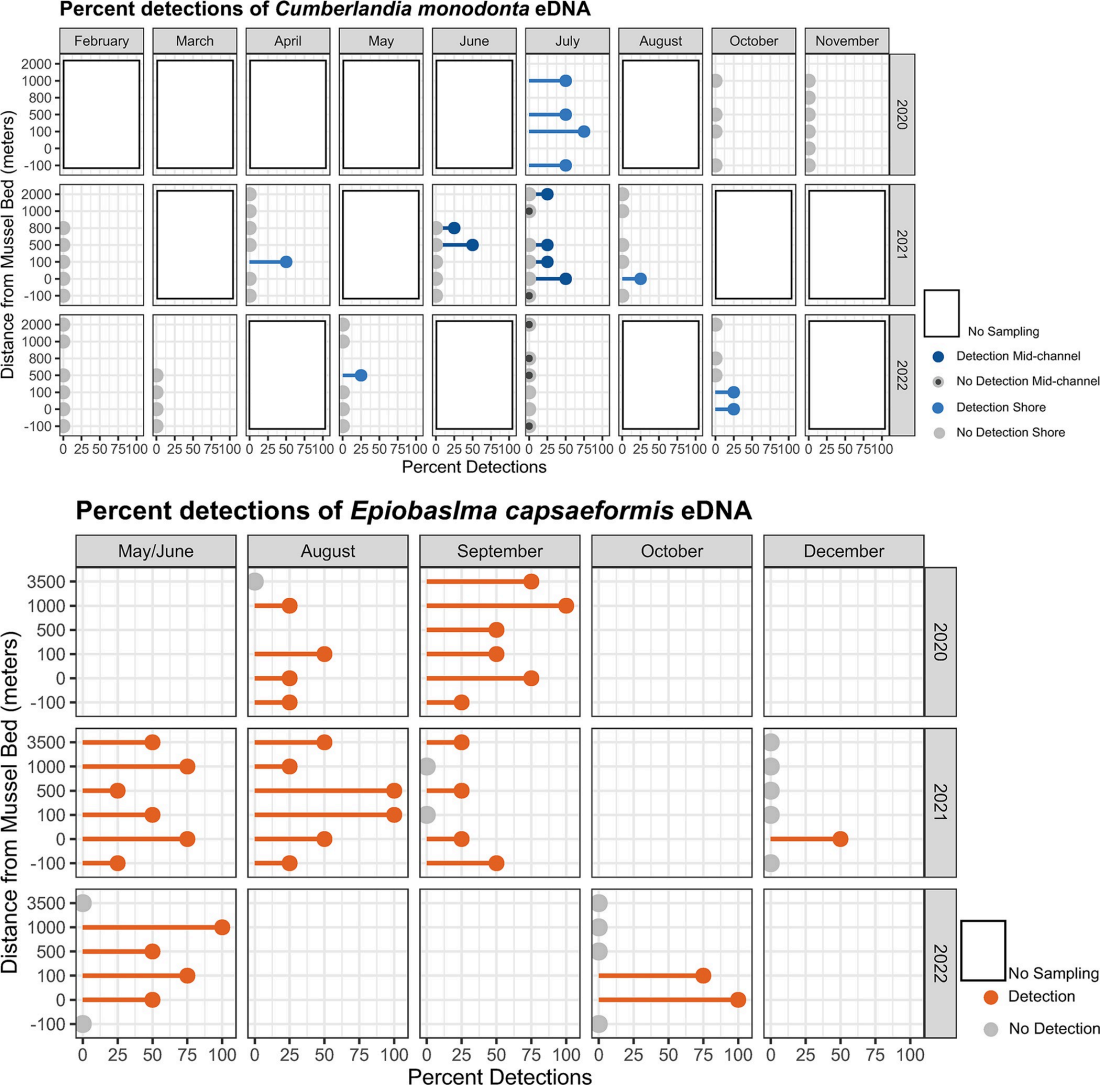
Environmental DNA detections. Percent detections for *Cumberlandia monodonta* in the Lazy Day reach in the Big Piney River, MO (A) and for *Epioblasma capsaeformis* in the Wallens Bend reach in the Clinch River, TN (B).

Environmental DNA was sampled across 13 sample events at Lazy Day and 9 sample events at Wallens Bend (Table 1, Fig A in S1 Text). In total, we collected 330 field samples, 84 field blanks, and 33 extraction blanks from Lazy Day and 188 field samples, 47 field blanks, and 36 extraction blanks from Wallens Bend. For Lazy Day, all extraction blanks were negative for amplification, and only 1 field blank showed amplification. This field blank was at the 100 m upstream stretch of Lazy Day, and the four field samples associated with it showed no amplification. For Wallens Bend, 1 extraction blank at the 100 m upstream site amplified, but no amplification occurred in the four associated field samples. All extraction blanks were clean. We were able to detect eDNA from both target species, but mean detection rates at each river were low (Lazy Day: *C*. *monodonta* = 6.25%; Wallens Bend: *E*. *capsaeformis* = 36.7%; Fig 2;Tables D and E in S1 Text). At Lazy Day, the average of the four replicate field samples ranged 0 to 197 copies/L for *C*. *monodonta*, but only one replicate was above the assay’s limit of quantification and only two replicates (<1%) were above the limit of detection. At Wallens Bend, the average of the four replicate field samples ranged from 0 to 344 copies/L for *E*. *capsaeformis*. Here, no replicates were above the limit of quantification, but 12 (6.4%) were above the limit of detection. There were no inhibited samples from Lazy Day, but four samples from Wallens Bend showed IPC inhibition.

Field sample eDNA detections and concentrations varied across and within sampling events and at sampling locations within each study reach. It was common to observe differences in eDNA detection across the four replicate samples at a sampling location as well as a large range in eDNA concentration. Within each study reach, eDNA concentrations generally decreased farther downstream from the mussel bed. Moreover, seasonal trends were also not apparent. For example, *E*. *capsaeformis* eDNA concentration downstream from the mussel bed at Wallens Bend was detected in only 25% of the total samples in August of 2020 (0 to 98 copies/L), but 58% of the samples in August of 2021 (0 to 344 copies/L; Fig 2B). In Lazy Day, we did observe differences in both detection and concentration between the mid-channel and shore samples. *Cumberlandia monodonta* eDNA was detected in two of the three sampling events where mid-channel samples were taken with an average detection probability of 12.5 and 37.5%. None of the shore samples detected eDNA during either of these two sampling events with mid-channel detections.

### 3.3. Hydraulic modeling

The hydraulic model for Lazy Day was calibrated using five calibration discharges ranging from ~8 to 64 m^3^/s. The model for Wallens Bend was calibrated using four discharges from ~14 to 43 m^3^/s. The model calibrations yielded best fit regression relationships (calibration equations) between discharge and several variable model parameters. For Lazy Day, the variable model parameters were downstream water-surface slope and Manning’s *n*; for Wallens Bend, the variable model parameters were Manning’s *n* and the upstream water-surface elevations in the Clinch and North Fork. The calibration equations for each model reach are provided in USGS ScienceBase data releases [32,33]. Calibrated Manning’s *n* values ranged from 0.0275 to 0.0395 for Lazy Day and 0.03 to 0.06 for Wallens Bend.

**Fig 3:**
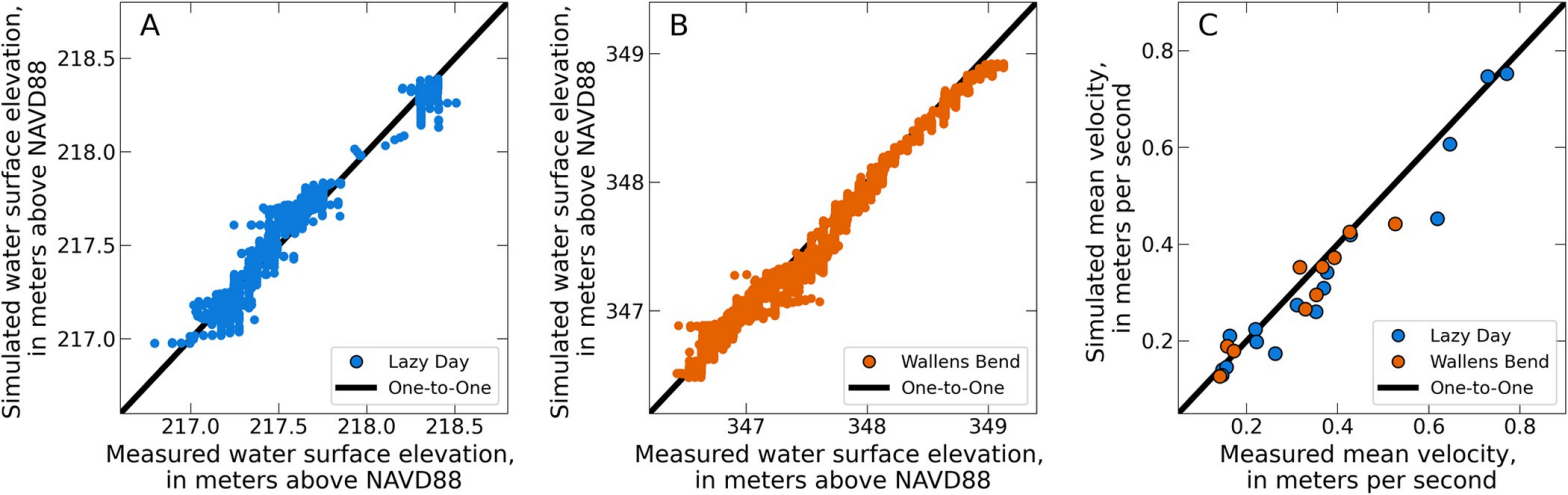
Hydraulic model calibration. The hydraulic models at each study reach were calibrated to the measured water-surface profiles, and model performance was evaluated using the mean velocity at select transects. The hydraulic model performance for the water surface profile for all calibration flows is shown in panels A and B. Likewise, the hydraulic model performance for water velocity is shown for all calibration flows in C.

Model calibration performance was evaluated for each of the calibration discharges using qualitative and quantitative methods. Qualitative evaluation was performed by visually comparing the computed average velocity at ADCP measurement locations with the simulated average velocity (Fig 3). Quantitative evaluation was performed using the RMSE between the measured and simulated average velocities. The maximum RMSEs for measured and simulated average velocity values for Lazy Day and Wallens Bend were 0.12 m/s and 0.06 m/s, respectively. The maximum RMSEs for measured and simulated water-surface elevation for Lazy Day and Wallens Bend were 0.086 m and 0.105 m, respectively.

The sensitivity analysis for Lazy Day yielded maximum differences in simulated water-surface elevation and velocity of 0.207 m and 0.476 m/s, respectively. The sensitivity analysis for Wallens Bend yielded maximum differences in simulated water-surface elevation and velocity of 0.135 m and 0.282 m/s, respectively. Overall, these analyses show that the variation in simulated velocity and water-surface elevation is minimal and indicate that uncertainties in the Manning’s *n* calibration do not have a strong effect on the hydraulic model results.

### 3.4. eDNA transport modeling

Transport simulations were based on measured eDNA concentrations, the calibrated hydraulic model, and the default calculation of the longitudinal dispersion coefficient, *D*. Only two of the sampling events for Lazy Day (July 07, 2020 and July 23, 2021) had sufficient detections throughout the study reach to simulate eDNA. Six sampling events did not detect *C*. *monodonta* in Lazy Day. Five other sampling dates did detect *C*. *monodonta* in Lazy Day, but eDNA detections from these dates were intermittent throughout the reach or occurred at only one or two sampling locations downstream from the mussel bed, and thus insufficient to simulate in a transport model. Three of the sampling events at Wallens Bend had sufficient detections to simulate eDNA transport. Environmental DNA was detected during the other six sampling dates, but either intermittently downstream from the mussel bed or with higher concentrations further downstream compared to the concentration at the mussel bed; data were also insufficient to simulate a transport model.

**Fig 4:**
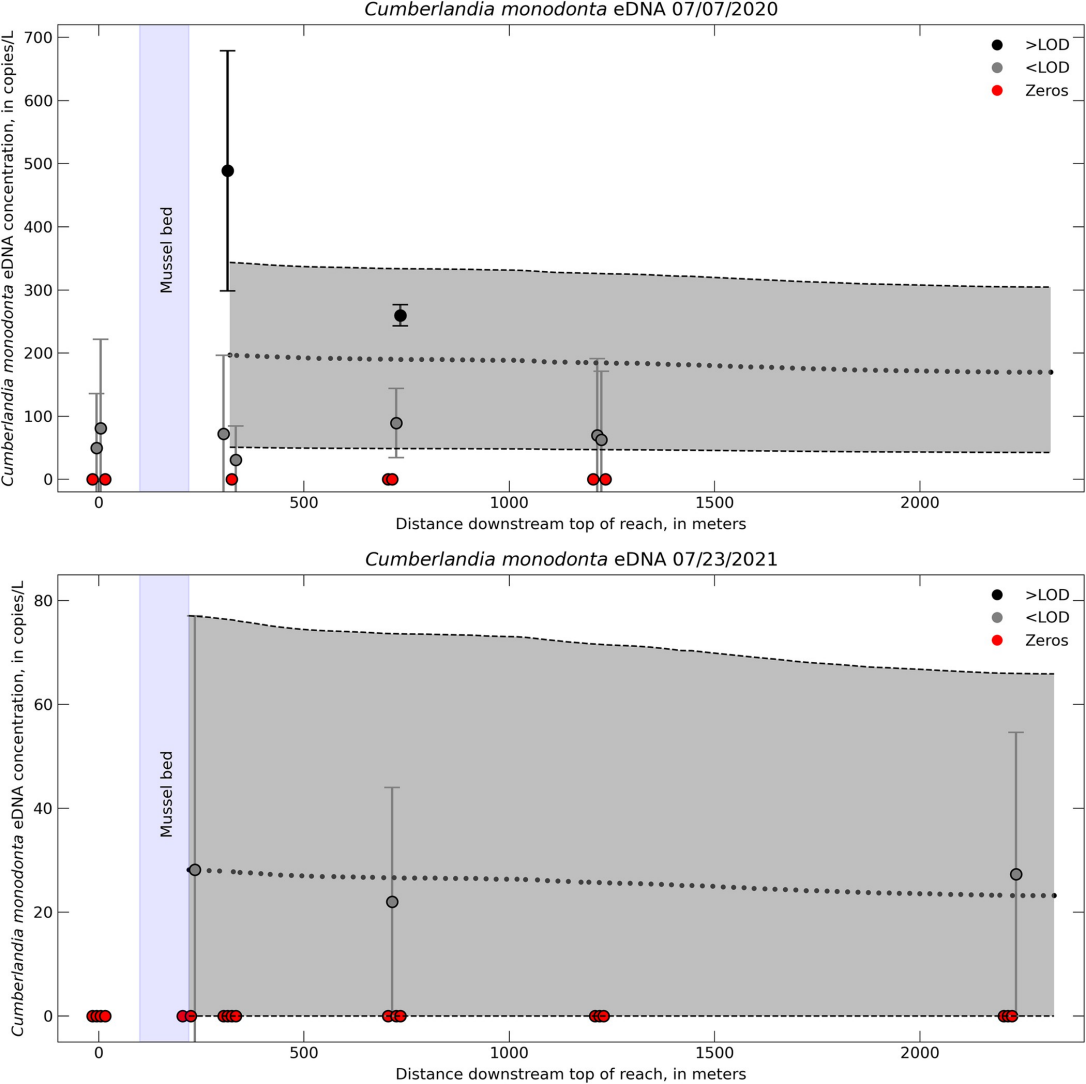
Environmental DNA transport model for the Lazy Day reach. One-dimensional transport model eDNA concentrations (dotted black line) compared to field sampled eDNA concentration for *Cumberlandia monodonta* in the Lazy Day reach in the Big Piney River, MO. Different colors indicate samples either above or below the limit of detection (LOD) or samples where no eDNA was detected (zeros). Error bars on the field samples represent ± 1 standard error (SE) from the qPCR triplicates. Error associated with the model includes ± 1 SE of the initial eDNA concentration and the decay rate, which is represented by the gray shaded region.

**Fig 5:**
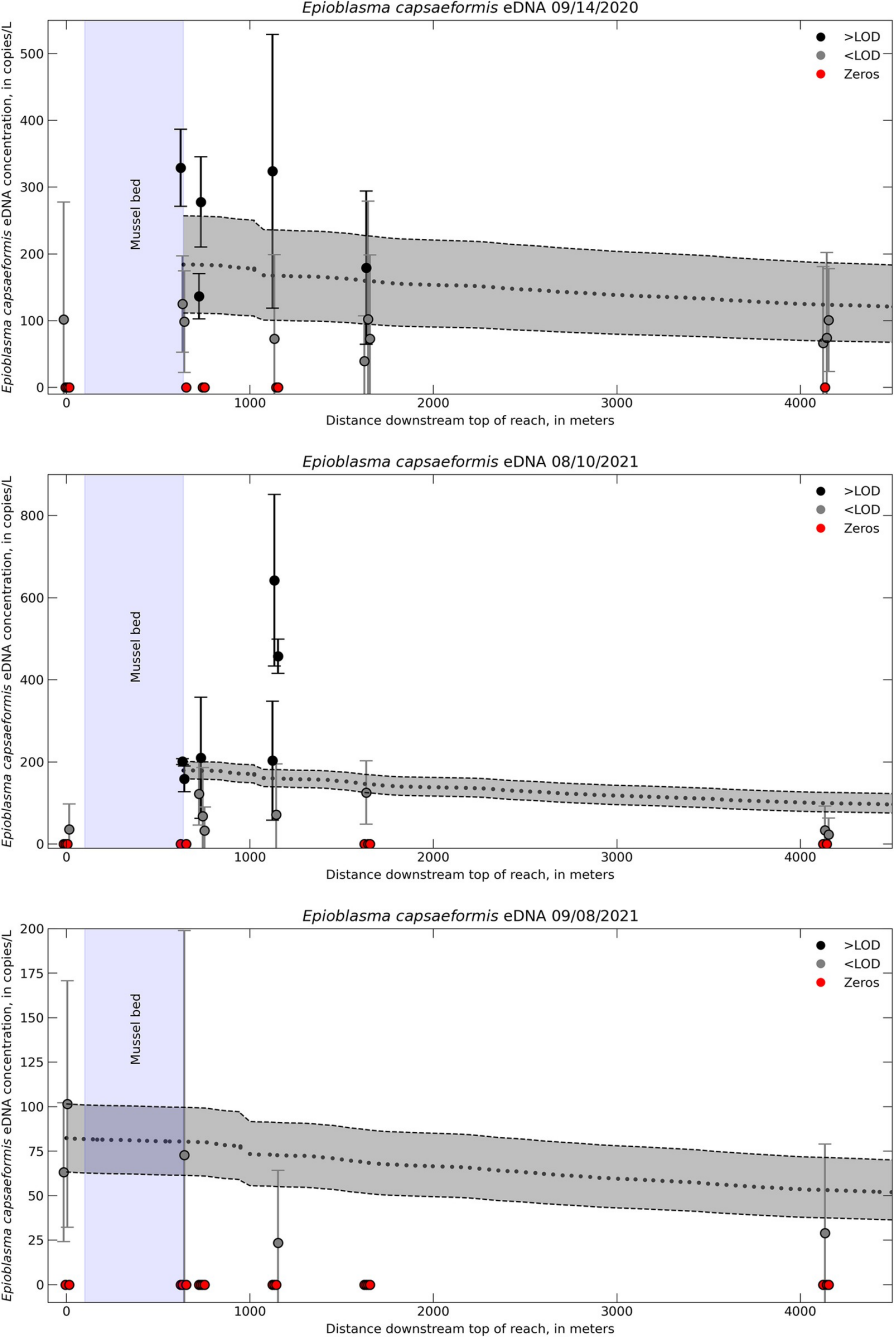
Environmental DNA transport model for the Wallens Bend reach. One-dimensional transport model eDNA concentrations (dotted black line) compared to field sampled eDNA concentration for *Epioblasma capsaeformis* in the Wallens Bend reach in the Clinch River, TN. Different colors indicate samples either above or below the limit of detection (LOD). Error bars on the field samples represent ± 1 standard error (SE). Error associated with the model includes ± 1 SE of the initial eDNA concentration and the decay rate, which is represented by the gray shaded region.

Simulated eDNA concentrations declined as a function of distance downstream (Figs 4 and 5). The downstream rate at which eDNA concentrations changed was influenced by hydraulic and geomorphic features in each river. For example, eDNA was quickly transported through riffle-run reaches (i.e., slower decline in concentration) but moved more slowly in pools (i.e., faster decline in concentration). One notable feature at Wallens Bends was the North Fork tributary. Simulated eDNA concentrations had a high rate of decrease immediately downstream from the confluence with the North Fork and Clinch Rivers (around 1,000 m downstream top of reach on Fig 5).

**Fig 6:**
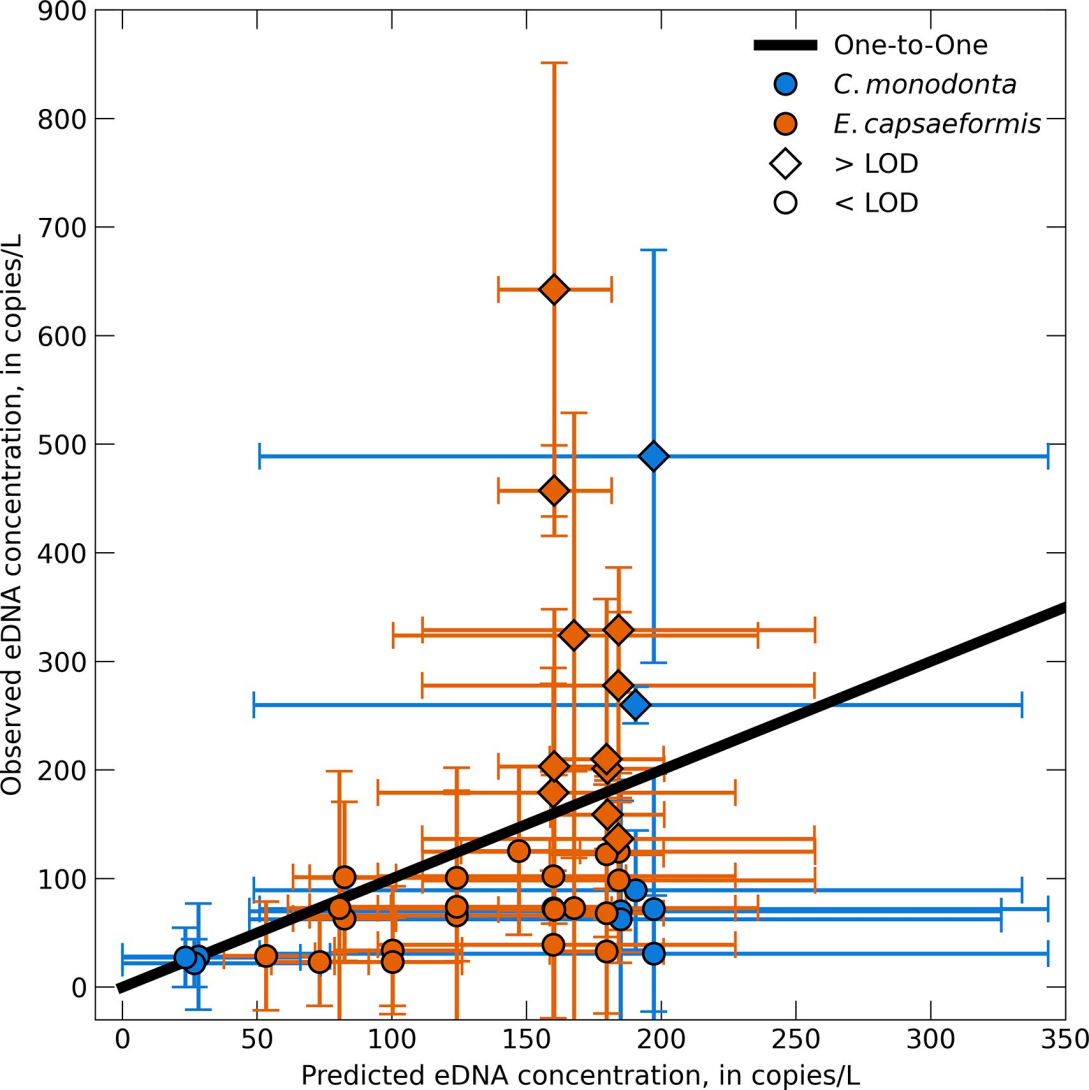
Model predictions compared to eDNA field samples. The predicted model concentrations compared to the field sampled eDNA concentrations for *Cumberlandia monodonta and Epioblasma capsaeformis*. The black line represents a one-to-one relationship. Horizontal error bars represent the upper and lower error estimates from the model, while the vertical error bars represent ± 1 standard error (SE) of the qPCR triplicates.

Overall, the model predictions were generally within ± 1 SE of the field sample eDNA concentrations (Figs 4–6). We were able to incorporate the variability of the eDNA concentration of the field samples into our model by taking an upper and lower bound that represented ± 1 SE of the mean field sampled eDNA concentration at the upstream boundary condition as well as ± 1 SE of the decay coefficient. However, doing so also resulted in a relatively large range of modeled eDNA concentrations at a given distance downstream from the transport model. This level of sensitivity captures the variability within the model attributed to biological uncertainties associated with eDNA degradation or variability across field sample concentrations, but does not necessarily capture other biotic and abiotic uncertainties that led to high variability in the field samples.

### 3.5. Statistical analyses

None of the linear regressions between eDNA concentration and discharge, water temperature, conductivity, or pH were statistically significant (Table 1). The strongest correlations were between water temperature and eDNA concentration at Wallens Bend (R^2^ = 0.36) and pH and eDNA concentration at Lazy Day (R^2^ = 0.27). Both of these showed a slight increase of eDNA concentration with increasing temperature or pH. All remaining regressions had less than 0.11% correlation to eDNA concentration and had no discernable trends.

## 4. Discussion

Our study aimed to detect eDNA from two federally endangered mussels and develop a transport model using field based eDNA concentrations and laboratory derived eDNA decay coefficients, and to incorporate geomorphic and hydraulic characteristics of each study reach to better understand downstream transport of eDNA. We successfully detected eDNA from both mussel species, although detection probability was generally low and varied greatly in both time and space. Field sampled eDNA concentrations were also highly variable within and across rivers and sampling locations. The transport models captured the variability of eDNA concentration associated with the field samples, had relatively strong agreement with the eDNA concentration from field samples at locations downstream from each mussel bed, and also showed a similar trend of decreasing eDNA concentration moving downstream from the mussel bed. Overall, this study demonstrated the ability of eDNA methods to detect sensitive target organisms and provided a hydraulic transport model to predict the downstream transport of eDNA.

### 4.1. eDNA field samples

Use of eDNA quantification estimates is important for studying the processes that lead to changes in eDNA signal. These processes are collectively known as eDNA ecology [18] and include eDNA production or shedding, degradation, adsorption, and transport. However, important questions need to be addressed in best practices for reporting eDNA detections and concentrations below an assays LOD and LOQ. The LOD of an assay is used to ensure that an eDNA qPCR-based assay has acceptable sensitivity, because the LOD represents the concentration below which there is a greater than 5% chance of a false negative, or failure to detect the target sequence even though it is present in the sample. Values that fall below the LOD are still considered true amplifications, whereas the LOQ provides a level of precision, usually set at 35% coefficient of variation, at which concentrations can be estimated [36]. Detections below the LOQ have lower than the desired precision in quantifying the amount of starting DNA concentration. As in the present study, we often observed eDNA field detection concentrations below the assay LOD and LOQ despite using highly optimized and sensitive assays. It would be most informative if reported eDNA data would include all measured concentrations of samples, including zeros, and flag those below the assay’s LOQ. Deleting or substituting values below the LOD and LOQ could result in biased concentrations [45]. In current practice, most papers do not provide the number of samples below LOD/LOQ, although most now do report the assay’s LOD and LOQ values. This information is important when reporting concentrations that will be used for further calculations or inferences, particularly those involving transport modeling. Improved methods of quantification that will lower the effective LOD and LOQ are an active area of eDNA research [46]. Measured values below the LOD and LOQ still represent the best available estimate of the sample concentration; these values can be used for quantitative analyses of the data with the caveat that researchers report the precision of these estimates. Furthermore, it would be beneficial if eDNA studies include data on both sample concentrations and percent detections in order to better understand the properties of eDNA data at low concentrations and improve ability to analyze them.

The low eDNA concentrations were surprising given that the target species occur in dense aggregations and previous studies have recorded high eDNA detections downstream of large (100 mussels or more) mussel aggregations [46]. The *C*. *monodonta* bed at Lazy Day is a substantial single-species aggregation with average densities around 4.9 mussels m^-2^ and a population estimate of more than 4,000 animals in 2021 (A. Roberts, U.S. Fish and Wildlife Service, oral communication, September 2022). The mussel bed at Wallens Bend is a large and species-rich mussel bed. At the most recent survey in 2021, *E*. *capsaeformis* densities averaged >5 mussel m^-2^ with an estimated abundance of more than 55,000 individuals (C. Carey, Virginia Tech, oral communication, August 2022). In general, we did detect *E*. *capsaeformis* eDNA more often at Wallens Bend than we detected *C*. *monodonta* eDNA at Lazy Day, but the moderate detections and low concentrations observed in this study are of note because the populations of *C*. *monodonta* and *E*. *capsaeformis* at their respective beds are considered to be relatively large, especially for endangered species.

The use of eDNA to monitor and detect freshwater mussel populations is still being developed and results to date have varied. For instance, Currier, Morris [47] detected eDNA from four freshwater mussel species at all sites with positive visual identification from quadrat sampling. Deiner and Altermatt [21] detected eDNA from *Unio tumidus* (Retzius, 1788) at 9 km downstream from a known population and Stoeckle, Beggel [48] detected eDNA from *Unio crassus* (Retzius, 1788) 3.2 km downstream from mussel beds in five different streams. On the other hand Schmidt, Spear [49] did not detect *Lasmigona decorata* (Lea, 1852) eDNA at any sites known to harbor this species in the field and suggest that low-density mussel populations will be difficult to detect with current eDNA methods. Interestingly, Lor, Schreier [34] had similar low detection rates of *C*. *monodonta* in the St. Croix and Upper Mississippi Rivers compared to our study, but no detections were above their *C*. *monodonta* assay’s LOQ.

The low detection rates observed in our study could be attributed to a number of factors including but not limited to season, river hydraulics and geomorphic features, and mussel habitat and physiology. We designed our field sampling strategy to monitor changes in eDNA detection and concentration across multiple seasons, potential spawning events, hydraulic characteristics, and environmental variables, but the overall low detections in both rivers made it difficult to statistically link eDNA detections and concentrations to biotic or abiotic variables (Fig B in S1 Text). Our results from the eDNA field samples, however, do indicate several key considerations for studies using eDNA to detect and discern species distribution, which we highlight below.

The spatial location laterally across a river appears to influence both eDNA detections and concentrations. Two of our sampling events at Lazy Day where we collected samples from both near the shore and the mid-channel only detected eDNA at the mid-channel locations. Similarly, Stoeckle, Beggel [48] observed high detections with samples taken from the middle of each stream and Shogren, Tank [14] reported generally higher eDNA concentrations from samples taken at the center of the river compared to those taken from the side. Moreover, mussel physiology and associated habitat likely limit the amount of eDNA available in the water column. Because mussels are benthic and relatively sedentary organisms, much of the DNA exuded is released into the sediment or very near the water-sediment boundary. As a result, much of the eDNA released by mussels may quickly become trapped in the sediment [see 14,23] or may never fully mix into the water column [see 20,50]. Benthic samples offer the potential to improve detection, but studies are limited and so far indicate conflicting results on the benefit of improved detections using benthic eDNA samples [34,47]. Additionally, mussels are enclosed in a calcified shell that protects their soft tissues that may further limit the amount of DNA released into the environment. Indeed, Andruszkiewicz, Zhang [51] hypothesized that a lower eDNA shedding rate in shrimp compared to fish was because the hard exoskeleton of shrimp limited the amount of eDNA shedding. Studies reporting eDNA shedding rates for freshwater mussels are limited and the reported rates vary by orders of magnitude [11,29,52,53]. The low detections observed in our study and other studies with freshwater mussels [29,34,49], conflicting and limited evidence to support the optimal location for eDNA samples, and the limited understanding on how much DNA is shed from mussels highlights two important research needs related to detecting freshwater mussel eDNA: a) the need to optimize the spatial distribution of eDNA sampling locations, including the distance to sample downstream from a mussel bed, where to sample laterally across a river, and whether samples should be taken in the water column, and location in the water column, or substrate, and b) the need to improve understanding of species-specific eDNA shedding rates for freshwater mussels.

### 4.2. eDNA transport modeling

The hydraulic models for both Lazy Day and Wallens Bend were well calibrated to the field collected hydraulic data (Fig 3), which provides high confidence for replicating key hydraulic and transport processes within the model for the range of calibrated flows. Despite our assay’s low LOD and LOQ, most of our eDNA field samples were still below these values. Nevertheless, we decided to compare our model predictions with the measured concentration values even though they are below the LOQ, because the measured values were the best available estimates of the target eDNA concentrations. Overall, the predicted eDNA concentrations from the transport model were within ±1 SE of the eDNA concentrations from the field samples, indicating the ability of the model to predict downstream concentrations of eDNA from a source population. Variability in the model predictions was high, but this was caused by the high variability in the field eDNA concentrations used to initialize the model. Improvement in field-based detections and concentration estimates would help reduce the uncertainty in the model predictions. For example, there were several instances where the model underpredicted the field observations at concentrations greater than 150 copies/L. All of these were values above the assay’s LOD, which indicates bias in our models towards concentrations less than the LOD that were used as the upstream boundary conditions (Fig 6). Moreover, the small differences in eDNA concentration across the entire study reach may not reflect a true detectable difference in eDNA field samples, as indicated by the high variability of eDNA field samples and model predictions. Because of this, it may be challenging to derive net eDNA transport distances using 1D hydraulic models with concentrations below the LOD. Despite the variability in the model predictions and at concentrations below LOD, the 1D hydraulic transport models demonstrate the potential as a predictive tool to estimate eDNA concentrations downstream from source populations and provide a framework to guide eDNA field sampling strategies.

We hypothesized that integrating geomorphic and hydraulic features into a 1D hydraulic model would improve upon numerically based models [see 11–13] and reduce the computational time needed to perform multidimensional modeling [see 15]. However, because our eDNA transport models were simulated in only one dimension (i.e., longitudinal), spatial trends in eDNA concentrations were not resolved laterally across the stream or vertically through the water column and may have contributed to some of the disconnect between simulated eDNA concentrations and field-sampled eDNA concentrations. For example, the model hydraulics were calculated at evenly spaced cross sections throughout each study reach, but only a single hydraulic variable (i.e., water velocity, water depth, etc.) is calculated as an average for each cross section. Conversely, eDNA field samples were taken at point locations ~1 to 2 m off the shore (about 1 to 3 percent of the river width depending on discharge) near the water surface (or mid-channel near the water surface for select sampling events). Because eDNA may stay within streamlines and not mix evenly as it is advected downstream [20,50], it is possible that eDNA collected in field samples farther upstream remained in a narrow plume and was not dispersed to subsequent sampling locations farther downstream. We did expect, however, that our downstream sampling locations (1000, 2000, and 3500 m downstream the mussel bed) were appropriately spaced to capture eDNA through a ‘breakout phase’ where particle fragmentation and turbulent mixing results in more evenly distributed concentrations and perhaps that eDNA at our most downstream sampling locations may persist at higher concentrations near the banks [54].

Incorporating additional complexities, such as depositional velocity of eDNA, sediment interactions, or hydrogeomorphic features including pools or riffles, may also improve model predictions, but our eDNA field data did not support such inclusion. Depositional velocities (*v*_*dep*_) ranging from 0.15 to 0.54 mm s^-1^ have been reported [23,24], but the approaches used to estimate these rates place a strong emphasis on using a mean water velocity or stream depth, thereby stressing the importance of including other hydraulic factors at a higher spatial and temporal resolution. Growing evidence also indicates that sediment interactions with eDNA are important and could be included in future modeling efforts. Fremier, Strickler [7] observed that modeled eDNA concentrations were about three times higher than observed concentrations and concluded that passive retention of eDNA in biofilms and other streambed surfaces results in transient storage that was not captured in their model. Similarly, Shogren, Tank [14] estimated that physical retention of eDNA accounted for 50 to 80% of eDNA removal compared to biological degradation. Moreover, there is likely a delayed response between eDNA transport and water advection because of repeated cycles of adsorption and resuspension [9,23].

## 5. Conclusions

We detected eDNA across several seasons and flow rates for two federally endangered mussels in two geographically distinct rivers. There was, however, substantial variation in both the detection rate and eDNA concentration across and within sampling events, rivers, and sampling stations and the increased uncertainty in qPCR measurements of target species eDNA complicated the data analysis and interpretation for the present study. Using the concentration from eDNA field samples, along with hydraulic and geomorphic characteristics of each study section, we developed a 1D hydraulic transport model to predict the fate and transport of eDNA in lotic environments. While additional biophysical processes such as eDNA depositional velocity or interactions between eDNA and sediment are likely to improve our modeling framework, additional improvements to the model cannot be made with the observed level of detection and eDNA concentrations. This study highlights the need to optimize the spatial locations where eDNA is collected downstream from a source population, improve understanding on the mechanisms and magnitude of eDNA shed from source populations, and incorporate appropriate biogeomorphic processes into eDNA transport models.

## Supporting information

S1 Text Supporting information. Supplemental information includes additional detail on design, specificity, and sensitivity for each assay, detail on model development, and additional results.
(DOCX)
