# Supplementary material for: Detection and transport of environmental DNA from two federally endangered mussels
Source: PLoS One. 2024 Oct 17;19(10):e0304323. doi: 10.1371/journal.pone.0304323 (PMC11486370; doi:10.1371/journal.pone.0304323)
Supplement: S1 Text — Supplemental information includes additional detail on design, specificity, and sensitivity for each assay, detail on model development, and additional results. (DOCX) [file pone.0304323.s001.docx]

# Supporting Information 1: Text

## Assay design

| Table A: Assay information. Assay name, target gene for amplification, oligo type and name, oligo sequence, length of the amplified product, limit of detection (LOD) for the lowest standard tested with 95% or greater positive detections (Low.95) and modeled for 3 replicates (3REPS), and limit of quantification (LOQ), and efficiency of the assay. | | | | | | | | | |
| --- | --- | --- | --- | --- | --- | --- | --- | --- | --- |
| Assay | Target Gene | Oligo | Name | Sequence | Amplicon length | LOD (Low.95) | LOD (3 REPS) | LOQ | Efficiency |
| E.cap.COI3 | CO1 | Primer1 | E.cap.CO1-F3 | GGACAGCCAGGTAGGTTATTG | 106 | 4.5 | 1.43 | 12 | E= 98% |
|  |  | Primer2 | E.cap.CO1-R3 | CACCAATCATCATCGGCATTAC |  |  |  |  |  |
|  |  | Probe1 | E.cap.CO1-P3 | TGTGATTGTGACGGCACATGCTTT |  |  |  |  |  |
| M.mon.COI2 (Lor, Schreier, Waller, & Merkes, 2020) | CO1 | Primer1 | MmonCOI-F2 | GTTACGATTACGGCAGTTTTGCT | 119 | 8 | 1.39 | 18 | E = 95% - 110% |
|  |  | Primer2 | MmonCOI-R2 | TCACCCCCACCGGTAGGA |  |  |  |  |  |
|  |  | Probe1 | MmonCOI-PR2 | TGGTGCTATTACTATGTTATTGACCGATCG |  |  |  |  |  |

## Assay specificity and sensitivity

| Table B: Species tested against the *Cumberlandia monodonta* CO1 assay. Scientific name of the species tested, whether the species is sympatric (occurs in same location) as *C*. *monodonta*, and whether the sample was amplified with the assay. | | |
| --- | --- | --- |
| Species tested | Sympatric | Amplified |
| *Ortmanniana ligamentina* * | YES | NO |
| *Ortmanniana pectorosa* * | NO | NO |
| *Alasmidonta marginata* | YES | NO |
| *Cumberlandia monodonta* | N/A | YES |
| *Eurynia dilatata* * | YES | NO |
| *Epioblasma brevidens* | NO | NO |
| *Epioblasma capsaeformis* | NO | NO |
| *Epioblasma f. aureola* | NO | NO |
| *Fusconaia cuneolus* | NO | NO |
| *Fusconaia subrotunda* | NO | NO |
| *Hemistena lata* | NO | NO |
| *Lampsilis ovata* | NO | NO |
| *Lasmigona costata* | YES | NO |
| *Lemiox rimosus* | NO | NO |
| *Pleuronaia dolabelloides ** | NO | NO |
| *Ligumia recta* | YES | NO |
| *Plethobasus cyphyus* | YES | NO |
| *Pleurobema rubrum/Pleurobema sintoxia* | YES | NO |
| *Ptychobranchus fasciolaris* | NO | NO |
| *Leaunio vanuxemensis ** | NO | NO |

* Indicates recent taxonomic change

| Table C: Species tested against the *Epioblasma capsaeformis* CO1 assay. Scientific name of the species tested, whether the species is sympatric (occurs in same location) as *E. capsaeformis*, and whether the sample was amplified with the assay. * a non-Unionid species. | | |
| --- | --- | --- |
| Species tested | Sympatric | Amplified |
| *Ortmanniana ligamentina* * | YES | NO |
| *Ortmanniana pectorosa* * | YES | NO |
| *Alasmidonta marginata* | YES | NO |
| *Amblema plicata* | YES | NO |
| **Corbicula spp.* | YES | NO |
| *Cyclonaias tuberculata* | YES | NO |
| *Cyprogenia stegaria* | YES | NO |
| *Eurynia dilatata* | YES | NO |
| *Epioblasma brevidens* | YES | NO |
| *Epioblasma capsaeformis* | N/A | YES |
| *Epioblasma f. aureola* | YES | YES |
| *Epioblasma triquetra* | NO | YES |
| *Fusconaia cor* | YES | NO |
| *Fusconaia cuneolus* | YES | NO |
| *Fusconaia subrotunda* | YES | NO |
| *Hemistena lata* | YES | NO |
| *Lampsilis ovata* | YES | NO |
| *Lampsilis siliquoidea* | NO | NO |
| *Lasmigona costata* | YES | NO |
| *Lemiox rimosus* | YES | NO |
| *Pleuronaia dolabelloides* | YES | NO |
| *Ligumia recta* | YES | NO |
| *Cumberlandia monodonta* | YES | NO |
| *Medionidus conradicus* | YES | NO |
| *Plethobasus cyphyus* | YES | NO |
| *Pleurobema plenum* | YES | NO |
| *Pleurobema rubrum/Pleurobema sintoxia* | YES | NO |
| *Ptychobranchus fasciolaris* | YES | NO |
| *Ptychobranchus subtentum* | YES | NO |
| *Pustulosa pustulosa ** | YES | NO |
| *Strophitus undulatus* | YES | NO |
| *Cambarunio iris ** | YES | NO |
| *Leaunio vanuxemensis* * | YES | NO |

* Indicates recent taxonomic change

## One-dimensional hydraulic model development

## Field data collection

### Physical habitat data

Physical habitat data to support model development were collected using real-time kinematic (RTK) global navigation satellite system (GNSS) survey methods with a single base. All data used were collected in RTK-fixed integer solution status. A temporary benchmark was established at each reach using a Trimble R8s base receiver and a Trimble TDL 450H series RTK broadcast radio. Each temporary benchmark was established by logging with the base station system for at least 4 hours. Logged benchmark coordinates were corrected with static postprocessing using the National Geodetic Survey Online Positioning User Service (OPUS) (Mader, Weston, Morrison, & Milbert, 2003). These benchmarks were reoccupied and used for subsequent surveys, using the corrected coordinates. Overall benchmark root mean squared error (RMSE) values were 0.016 – 0.022 meter (m) for Lazy Day and 0.016 and 0.018 m for Wallens Bend.

Bathymetry data were collected along planned transects spaced either 2.5 m apart (Lazy Day upper reach: 100 m upstream of mussel bed to 1000 m downstream of mussel bed) or 50 m apart (Lazy Day lower reach; Wallens Bend entire reach). Data were collected using a CEE HydroSystems CEEPULSE 100 series survey grade single-beam echosounder mounted in a CEE HydroSystems CEE-USV Remotely Operated Survey Boat. Position data were acquired with a Trimble R2 GNSS Receiver mounted on top of the remote boat. Data were collected by driving the remote-controlled boat along the planned transects, transmitted to the field computer via Bluetooth, and logged using Hypack 2018-2020 survey software (Xylem, Inc.).

High-resolution terrestrial light detection and ranging (lidar) data were also collected at the Lazy Day reach in the Big Piney River to capture the topography of exposed, unvegetated banks and bars. The terrestrial mobile laser scanning system is mounted to a modular rail and includes a Velodyne LiDAR Puck LITE, an SBG Systems Ellipse2-D Inertial Motion Unit, and a Trimble R7 GNSS system with a Zephyr antenna. The Puck LITE was configured to record the last return and use 6 beams at 600 rotations per minute. Data were collected by securing the lidar rail system to a motorized boat and recording data while driving or paddling in the upstream or downstream direction through the study reaches. Data were logged using the Hysweep survey software in Hypack 2018-2020 (Xylem Inc.).

Additional bathymetry and topographic data points were collected in shallow or vegetated areas using a Trimble TSC3 handheld data controller and a Trimble R2 GNSS receiver mounted on a survey rod.

### Hydraulic model calibration and evaluation data

Hydraulic calibration data were collected at a range of discharge values to provide adequate data to simulate a range of unmeasured flow events. Hydraulic evaluation data were collected at a subset of discharge values to assess the performance of the calibrated model.

#### Discharge

Discharge data were collected using a 600-kilohertz RiverRay acoustic Doppler current profiler (ADCP; Teledyne RD Instruments) mounted in the CEE-USV remote boat (CEE HydroSystems) or off the bow of a motorboat. The ADCP measures depth and velocity as it moves across the channel, which together are used to compute discharge for each driven transect across the channel. Position data were collected using a Trimble R2 GNSS Receiver mounted directly above the ADCP. On each discharge measurement day, the ADCP compass was calibrated by slowly rotating the ADCP until the compass error reached 0.5 degree or lower. Discharge measurements were collected by driving the boat-mounted ADCP across the channel for at least four reciprocal transects at a single-thread location in the channel. Transects were driven back and forth until at least two transects in each direction were acquired, each with a discharge error of less than 5 percent (%) of the mean discharge (Mueller, Wagner, Rehmel, Oberg, & Rainville, 2009). Data were transmitted to the field laptop via Bluetooth and logged in WinRiver II software (Teledyne Marine).

#### Velocity

On several discharge measurement days, supplemental ADCP transects were driven at various locations through each study reach to provide velocity data for model evaluation. These data were collected using the same methods as the discharge data collection above but using a discharge error threshold of 10% of the mean discharge.

#### Water-surface profile

A water-surface elevation profile was collected for every discharge measurement. Position and water-surface elevation data were measured using a Trimble R2 GNSS receiver mounted on a motorized boat, remote boat, or survey rod, with a known offset to the water surface. Water-surface profile data were collected along the length of the study reach and recorded using Hypack 2018 – 2020 Survey software (Xylem, Inc.) or a Trimble TSC3 data controller.

Additional water-surface elevation data were provided by temporary stream gaging stations (gages) installed within the study reaches for the duration of hydraulic data collection (2020-09-22 to 2022-03-16 for Lazy Day; 2021-04-13 to 2021-09-09 for Wallens Bend). Two gages were installed at Lazy Day: one approximately 100 m downstream of the mussel bed, and one about 100 m downstream of the bottom end of the reach. Three gages were installed at Wallens Bend: one near 100 m upstream of the mussel bed, one about 275 m upstream of the bottom of the model reach, and one on a tributary, the North Fork, about 300 m upstream of the junction. The North Fork gage data were used for monitoring purposes only. Each gage was installed using ~15 feet of vented PVC pipe, secured to a stable tree near the water’s edge. Water level was logged every 15 minutes using an Onset HOBO MX2001 Water Level Data Logger installed in each gage. A vertically-referenced water level was measured for the gage location using the manual RTK methods described above. This elevation was used as the gage’s reference water level at deployment.

## Data Processing

### Elevation data processing

#### State aerial lidar data

Aerial lidar for the study areas was obtained from state GIS repositories to provide floodplain elevation data. Lidar for the Lazy Day reach (MO) (Quantum Spatial, 2018) were flown between December 1, 2017 and April 24, 2018 and obtained in raster Tag Image File format (TIFF) from the Missouri Spatial Data Information Service (MSDIS). Lidar for the Wallens Bend reach (TN) were flown between February 5 and April 4, 2016 and obtained in LAZ format (U.S. Geological Survey, 2017). The horizontal resolution for the MO lidar DEM is 0.5 m, and the point spacing for the TN LAZ data range from 1.056 to 1.216 feet.

Aerial lidar were filtered to ground points only, if necessary, and clipped to exclude areas covered by the bathymetry, manual RTK data, and terrestrial lidar.

#### Bathymetry data processing

Bathymetry data were manually edited in Hypack 2018-2020 single-beam editor (Xylem, Inc.). Points were deleted if they had erroneous elevations or if the GNSS position solution was not fixed. Edited bathymetry data were converted to an Esri shapefile.

To create a continuous DEM within the channel, bathymetry data were interpolated between transects in areas where transect spacing was greater than 2.5 m (Lazy Day) or 5 m (Wallens Bend). These supplemental data points provide the flexibility to build the 1D hydraulic model with straight transects, and to add additional transects if the model produces conveyance warnings due to an insufficient number of cross sections. Transects were interpolated using the XS Interpolation tool within the HEC-RAS Geometry Editor, at a spacing of 2.5 m for Lazy Day and 5 m for Wallens bend.

#### Terrestrial lidar processing

Terrestrial lidar data were manually edited in Hypack 2018-2020 MBMAX 64-bit HYSWEEP Editor (Xylem, Inc.). Points were removed if the GNSS position solution was not fixed, or if they appeared to represent non-ground surfaces such as vegetation or buildings. Edited lidar data were converted to LAS format.

The other elevation datasets (bathymetry, manual RTK data, and state aerial lidar data) were converted to LAS format if necessary and combined with the edited terrestrial lidar LAS. This combined dataset was filtered to remove nonground points using the Simple Morphological Filter (Pingel, Clarke, & McBride, 2013) in the Point Data Abstraction Library Python package (PDAL Contributors, 2018; Python Software Foundation, 2021). This method takes advantage of known ground elevations from the other datasets to filter nonground points in the terrestrial lidar. The filtered point data were clipped to the boundary of the terrestrial lidar data, thinned to a sampling distance of 2 m, and converted to a shapefile.

### DEM creation

The bathymetry, manual RTK data, terrestrial lidar, state aerial lidar, and interpolated bathymetry were converted to shapefile format and combined to create a TIN using Delaunay conforming triangulation. Soft breaklines were incorporated at the dataset boundaries to prevent artifacts between different datasets. TINs were manually edited based on expert opinion to remove artifacts caused by spatial gaps in data. These TINs were converted to a TIFF raster using natural neighbors interpolation, with a resolution of 2 m for both study reach DEMs.

The Lazy Day DEM raster contains a series of ponds near the downstream end; these were filled with an artificial elevation of 220 m (above bankfull) to prevent the model from filling them with water.

Because limited elevation data were collected in the side channel at Wallens Bend, the side channel bathymetry was estimated by extracting the DEM surface for the side channel wetted area from the state aerial lidar. These elevations represent an approximation of the water surface slope from water surface lidar returns. This sloped surface was compared with field-measured RTK bathymetry points from the side channel, and a representative offset was determined and subtracted from the sloped surface. This computed raster was mosaicked with the site’s TIFF raster to incorporate the estimated sloped channel surface. This workflow ultimately produces a trapezoidal channel within the side channel, with a bed slope that mimics the energy gradient of the water surface.

### Calibration data processing

ADCP data for model calibration and evaluation were processed using WinRiver II Software (Teledyne RD Instruments). WinRiver II uses the velocity and depth transect data to compute an average discharge for the driven transects and an average velocity for the measurement location. The computed average discharge with the lowest transect discharge errors was used as the final calibration discharge, and the computed average velocity data were used as evaluation data to evaluate the 1D model’s performance. ADCP measurements from different locations at Wallens Bend were used to compute discharge for either the North Fork or the portion of the Clinch reach upstream of the tributary, depending on where measurements were collected that day. ADCP transect data were also converted to shapefile format and snapped to a stream centerline to determine the streamwise location of each set of transect measurements.

Boat-collected water-surface elevation data were edited in Hypack 2018-2020 Single beam editor to remove erroneous and non-fixed position solution points. These edited data and the manual RTK water-surface data were converted to shapefile format and snapped to a stream centerline to determine the streamwise location of each point. Dense water-surface profiles (>1000 points) were thinned to one point per meter of streamwise distance, using the median value.

Water-surface elevation profiles were supplemented with continuous water-surface elevation data provided by the temporary gages. Water-surface elevations were extracted from the continuous gage records for each of the discharge measurement, using the closest timestamp.

## Hydraulic modeling

Our analysis uses 1D steady flow modeling to simulate the hydrodynamic conditions at the time and location of eDNA sampling events in the reaches. Our hydraulic modeling approach assumes that the study reaches did not experience appreciable topographic change from erosion and deposition during the period of investigation and over the range of simulated discharges.

### Model development

All 1D models were configured and run using HEC-RAS 5.0.7 (U.S. Army Corps of Engineers, 2019). The 1D models were configured to run at steady flow, with a normal depth boundary condition at the bottom of the reach. The slope was varied by discharge. All other program defaults were left unchanged. HEC-RAS model runs were performed with a custom script in Python 3.8.12 (Python Software Foundation, 2021), using rascontrol version 0.11 (Bannister, Brown, & Gutzmann, 2020). Rascontrol is a Python wrapper for the HEC-RAS Controller module for automating HEC-RAS (Goodell, 2014).

Model geometry was developed using the HEC-GeoRAS 10.5 extension (Esri, 2017) in ArcGIS Desktop 10.7.1. A 1D model was established for each reach using a stream centerline and spaced transects along the entire model reach. Cross sections were drawn perpendicular to flow and spaced approximately every 50 m, located on or near surveyed bathymetry transects. For all model transects, elevation data were extracted along the transect line from the study reach DEM.

The Lazy Day model has 94 transects and spans approximately 2,327 m between the most upstream and most downstream cross sections. Supplemental cross sections were placed between primary transects (resulting in ~25 m spacing) to mitigate potential conveyance ratio issues.

The Wallens Bend model consists of 101 transects on the main stem of the Clinch, 8 transects in the side channel, and 5 transects on the North Fork (approximate spacing 12m). The model reach length is approximately 4880 m between the upstream and downstream cross sections on the main stem. Due to the large size of the island in the reach, the side channel is modeled with a junction at the upstream and downstream ends. Flow is optimized at the upstream junction using split flow optimization. The North Fork is modeled as a tributary with a junction at the mouth with the Clinch River.

Specific configurations and model parameters for each study reach are described below; otherwise, all other program defaults were left unchanged.

The Lazy Day models were configured with a normal depth boundary condition at the bottom of the reach. Downstream slope was varied by discharge. For each model run, discharge was set to a single flow rate for the model domain.

The Wallens Bend models were configured with two boundary conditions: 1) known water surfaces at the upstream cross sections on the Clinch River and the North Fork, and 2) a normal depth boundary condition at the downstream cross section with the same slope used for all discharges. Because of tributary input from the North Fork and split flow around the island complex, multiple flow rates were used in the Wallens Bend model domain. The discharge for the North Fork was estimated at 5.5% of the main stem discharge downstream of the junction (measured or computed for 2021-04-14, 2021-04-15, 2021-09-08). The discharge for the side channel was estimated as 25% of the total discharge upstream of the junction; this value was adjusted during split flow optimization.

### Model calibration

Model calibration was performed for the range of calibration discharges using the discharge and water-surface profile data collected in the field. The purpose of calibration is to determine the appropriate model conditions for the simulation discharges, the discharges of interest that (in most cases) were not measured in the field. The calibrated model conditions were downstream slope (used for the normal depth boundary condition) and Manning’s roughness (Manning’s *n*) within the active channel. The Wallens Bend model also uses upstream water-surface elevations in the Clinch and North Fork as boundary conditions.

These calibrated values for the downstream slope and Manning’s *n*, and the measured values of upstream water-surface elevation (Wallens Bend only) were used to create a regression relationship between discharge and each parameter. These curves were generated by fitting power law or linear functions to the parameter values to find the optimal fit. These best fit regressions yield an optimized parameter value for each discharge value to be simulated.

### Model simulations

#### Flow simulation discharges

The simulation flows of interest are the study reach discharges at the time of eDNA sampling events (Fig. A1). Discharges within the study reach were calculated using the average ratio of the ADCP-measured discharges to the discharges recorded at the closest U.S. Geological Survey streamgage at the time of ADCP discharge measurements. To obtain the estimated discharge in the study reach for each day of eDNA sampling, this average ratio was multiplied by the U.S. Geological Survey streamgage discharge at the time of each eDNA sample collection. These estimated discharges were then averaged for each eDNA sampling day to get a single discharge for that day.

The estimated flow rates for Wallens Bend correspond to the discharge for the section downstream of the North Fork tributary. The tributary discharge was estimated as 5.5% of the main stem discharge downstream of the tributary junction. The main stem discharge upstream of the tributary was computed from the difference of these two flow rates.

Because all the eDNA samples were collected at low or moderate discharge, the simulation discharges do not exceed bankfull flow, nor do they extend beyond the active channel.

#### Simulations

Simulations were run using the optimized values for Manning’s n (both sites), downstream slope (both sites), and upstream water surface elevations (Wallens Bend) for each of the simulation discharges (see “Model Calibration”). Model outputs include average velocity (varying in the streamwise direction), and water-surface elevation through the study reaches.

#### Sensitivity analysis

Sensitivity analyses were performed for both the hydraulic model calibration and the transport model. Sensitivity analysis simulations were also performed using Manning’s n values at 85 and 115 % of the optimum calibrated Manning’s n. These sensitivity analyses were run for each of the simulation discharges to determine if varying the Manning’s n calibration by +/- 15 % has an appreciable effect on the simulated water-surface profiles and velocities. Similarly, the transport model can be sensitive to the longitudinal dispersion coefficient. Because we simulated a constant discharge and constant release of eDNA, the longitudinal dispersion coefficient in theory has a negligible influence on tracer concentration patterns (Fischer, 1979). However, we did explore the sensitivity related to the longitudinal dispersion coefficient, because in reality, neither discharge nor eDNA shedding is constant in time. Using 5 and 95 percent of the calculated dispersion coefficient at each water quality cell we found that the predicted eDNA concentration was generally close to the sensitivity of ± 1 SE of the decay constant. Given this, we assumed that the theoretical basis for the calculation of the longitudinal coefficient (Eq. 3) was sufficient for our modeling approach.


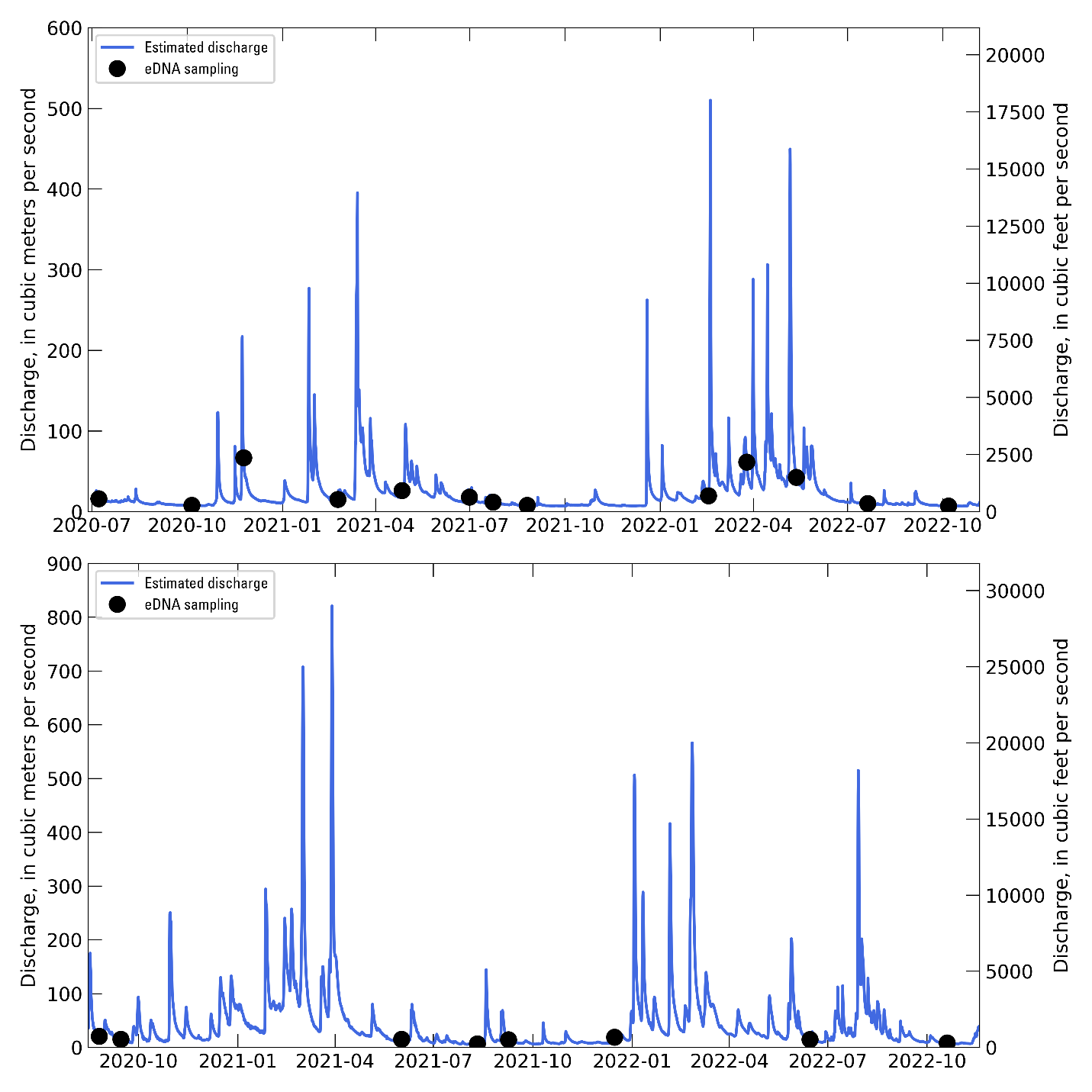


Fig A. The occurrence of eDNA field sampling and the estimated discharge in the Lazy Day reach in the Big Piney River, MO (top) and the Wallens Bend reach in the Clinch River (bottom) throughout the duration of our study.

## Results of eDNA field sampling

| Table D. List of dates, sample location, discharge values, and average eDNA detection percentage for *Cumberlandia* *monodonta* in the Lazy Day reach in the Big Piney River, MO. Bold values indicate the dates for which eDNA transport models were simulated. | | | |
| --- | --- | --- | --- |
| Date | Sample Location | Discharge (m3/s) | Mean Detection Rate (%) |
| **20200707** | **Bank** | **16.26** | **56** |
| 20201005 | Bank | 8.00 | 0 |
| 20201124 | Bank | 64.20 | 0 |
| 20210223 | Bank | 15.57 | 0 |
| 20210426 | Bank | 26.38 | 7 |
| 20210630 | Bank | 18.62 | 0 |
| 20210630 | Mid-Channel | 18.62 | 38 |
| 20210723 | Bank | 11.81 | 0 |
| **20210723** | **Mid-Channel** | **11.81** | **13** |
| 20210825 | Bank | 7.83 | 4 |
| 20220216 | Bank | 20.08 | 0 |
| 20220325 | Bank | 61.77 | 0 |
| 20220512 | Bank | 42.01 | 4 |
| 20220720 | Bank | 9.95 | 0 |
| 20220720 | Mid-Channel | 9.95 | 0 |
| 20221006 | Bank | 7.06 | 10 |

| Table E. List of dates, discharge values, and average eDNA detection percentage for *Epioblasma capsaeformis* in the Wallens Bend reach in the Clinch River, TN. Bold values indicate the dates for which eDNA transport models were simulated. | | |
| --- | --- | --- |
| Date | Discharge (m3/s) | Mean Detection Rate (%) |
| August 2020 | 21.8 | 25 |
| **September 2020** | **16.4** | **63** |
| May 2021 | 15.7 | 46 |
| **August 2021** | **7.3** | **58** |
| **September 2021** | **15.2** | **21** |
| December 2021 | 19.8 | 8 |
| June 2022 | 36.0 | 42 |
| October 2022 | 9.0 | 29 |


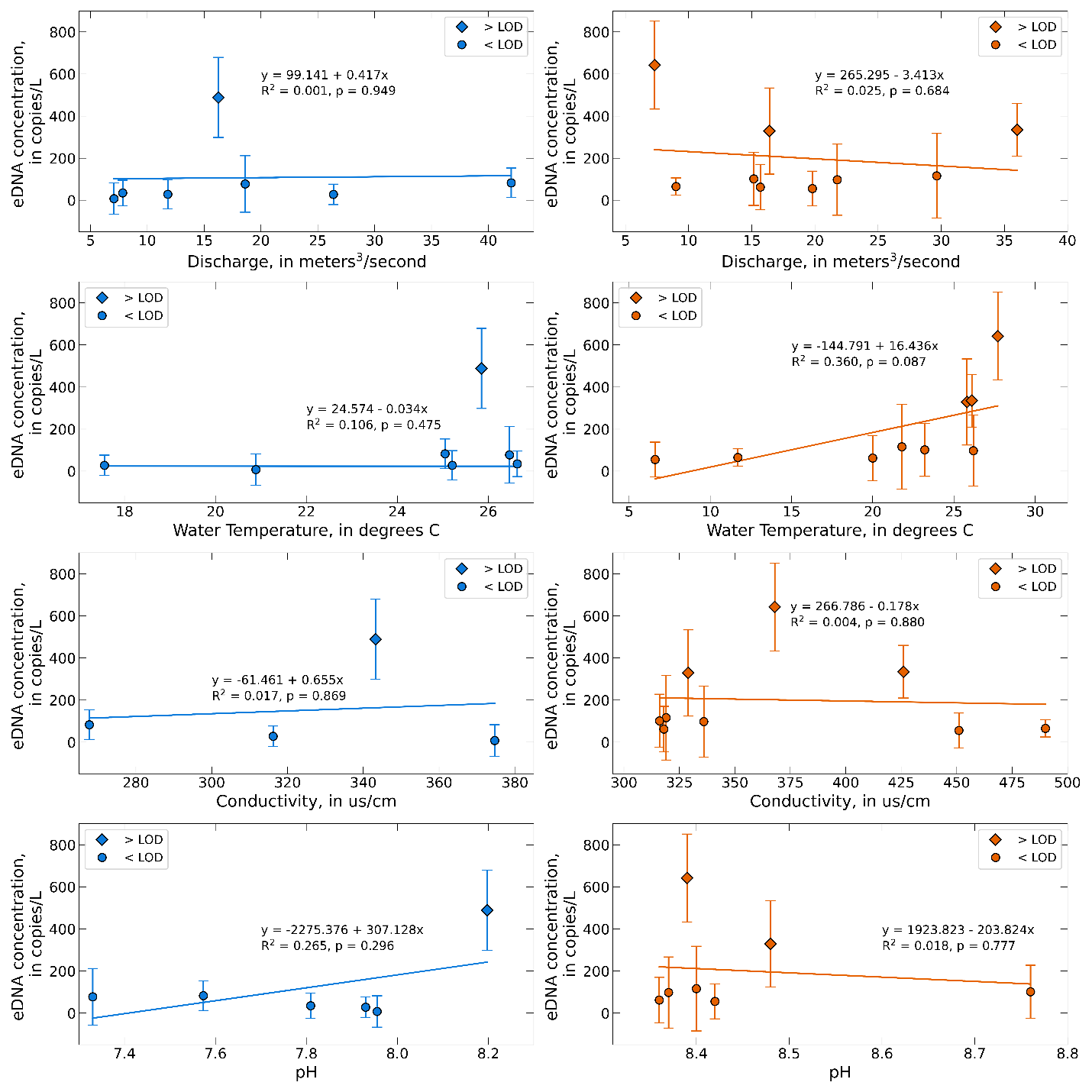


Fig B. The maximum environmental DNA concentrations for each sampling event for *Cumberlandia monodonta* in the Lazy Day reach in the Big Piney River, MO (left, blue symbols) and *Epioblasma capsaeformis* in the Wallens Bend reach in the Clinch River, TN (right, orange symbols) as a function of discharge, water temperature, conductivity, and pH. Error bars represent ± 1 SE of the qPCR triplicates.

## Supporting information references

Bannister, M., Brown, J., & Gutzmann, B. (2020). rascontrol (Version 0.11). Retrieved from <https://github.com/mikebannis/rascontrol>

Esri. (2017). HEC-GeoRAS 10.5. Retrieved from <https://downloads.esri.com/archydro/HecGeoRAS/>

Fischer, H. B. (1979). *Mixing in inland and coastal waters*: Academic press.

Goodell, C. R. (2014). *Breaking the HEC-RAS Code: A User's Guide to Automating HEC-RAS* (1 ed.). Portland, OR: h2ls.

Lor, Y., Schreier, T. M., Waller, D. L., & Merkes, C. M. (2020). Using environmental DNA (eDNA) to detect the endangered Spectaclecase Mussel (Margaritifera monodonta). *Freshwater Science, 39*(4), 837-847.

Mader, G. L., Weston, N. D., Morrison, M. L., & Milbert, D. G. (2003). The on-line positioning user service (OPUS). *Prof. Surv, 23*(5), 26-28.

Mueller, D. S., Wagner, C. R., Rehmel, M. S., Oberg, K. A., & Rainville, F. (2009). *Measuring discharge with acoustic Doppler current profilers from a moving boat*: US Department of the Interior, US Geological Survey Reston, Virginia (EUA).

PDAL Contributors. (2018). PDAL Point Data Abstraction Library (Version 1.8.0): Zenodo. Retrieved from <https://doi.org/10.5281/zenodo.2556738>

Pingel, T. J., Clarke, K. C., & McBride, W. A. (2013). An improved simple morphological filter for the terrain classification of airborne LIDAR data. *ISPRS Journal of Photogrammetry and Remote Sensing, 77*, 21-30.

Python Software Foundation. (2021). Python: A dynamic, open source programming language (Version 3.8.12): Python Software Foundation. Retrieved from <https://www.python.org/>

Quantum Spatial, I. (2018). Missouri 2017 LiDAR (Raster digital data). MSDIS Retrieved 8/31/2020, from Missouri Spatial Data Information Service, Washington University in St. Louis <https://arcg.is/15HqHz>

U.S. Army Corps of Engineers. (2019). HEC-RAS River Analysis System (Version 5.0.7). Davis, CA: US Army Corps of Engineers Hydrologic Engineering Center. Retrieved from <https://www.hec.usace.army.mil/>

U.S. Geological Survey. (2017). *Lidar Point Cloud (LPC)* [LAZ]. Retrieved from: <https://nationalmap.gov/3DEP/>
